# Eye movement analysis with hidden Markov models (EMHMM) with co-clustering

**DOI:** 10.3758/s13428-021-01541-5

**Published:** 2021-04-30

**Authors:** Janet H. Hsiao, Hui Lan, Yueyuan Zheng, Antoni B. Chan

**Affiliations:** 1 Department of Psychology, University of Hong Kong, Pok Fu Lam, Hong Kong; 2 The State Key Laboratory of Brain and Cognitive Sciences, University of Hong Kong, Pok Fu Lam, Hong Kong; 3 Department of Computer Science, City University of Hong Kong, Kowloon Tong, Hong Kong

**Keywords:** Eye movements, Hidden Markov model, Co-clustering, EMHMM, Scene perception

## Abstract

The eye movement analysis with hidden Markov models (EMHMM) method provides quantitative measures of individual differences in eye-movement pattern. However, it is limited to tasks where stimuli have the same feature layout (e.g., faces). Here we proposed to combine EMHMM with the data mining technique co-clustering to discover participant groups with consistent eye-movement patterns across stimuli for tasks involving stimuli with different feature layouts. Through applying this method to eye movements in scene perception, we discovered explorative (switching between the foreground and background information or different regions of interest) and focused (mainly looking at the foreground with less switching) eye-movement patterns among Asian participants. Higher similarity to the explorative pattern predicted better foreground object recognition performance, whereas higher similarity to the focused pattern was associated with better feature integration in the flanker task. These results have important implications for using eye tracking as a window into individual differences in cognitive abilities and styles. Thus, EMHMM with co-clustering provides quantitative assessments on eye-movement patterns across stimuli and tasks. It can be applied to many other real-life visual tasks, making a significant impact on the use of eye tracking to study cognitive behavior across disciplines.

**Supplementary Information:**

The online version contains supplementary material available at 10.3758/s13428-021-01541-5.

## Introduction

Eye-movement behavior has been shown to reflect underlying cognitive processes, and thus can potentially reveal individual differences in perception styles and cognitive abilities. For example, in scene perception, Chua et al. (2005) observed that Westerners made more eye fixations on foreground objects than Asians, whereas Asians looked at the backgrounds more often, and this difference was reflected in their object recognition performance (Masuda et al., 2016; Miyamoto et al., 2006). In face recognition, Peterson and Eckstein (2013) reported that individuals have different optimal viewing points, and deviation from this person-specific optimal viewing point impairs recognition performance, demonstrating the association between eye-movement patterns and task performance. People with cognitive deficits, such as neurodegenerative or psychotic disorders, have been reported to have atypical eye-movement patterns in visual tasks (e.g., Daffner et al., 1992), suggesting eye movements may be used for early detection of cognitive deficits.

Nevertheless, traditional approaches for analyzing eye-movement data, such as the use of predefined regions of interests (ROIs) on the stimuli (e.g., Barton et al., 2006) or the use of fixation heat maps/salience maps (e.g., Caldara & Miellet, 2011; Toet, 2011) do not adequately reflect individual differences in either spatial dimension (such as ROI choices) or temporal dimension (such as gaze transition among the ROIs) of eye movements. There have been attempts in using temporal information of eye movements in the analysis, such as using Levenshtein distance or sequence alignment algorithms to quantify and compare similarities of scan paths defined as a sequence of predefined ROIs visited (e.g., ScanMatch by Cristino et al., 2010; Goldberg & Helfman, 2010) or vector-based representations (Jarodzka et al., 2010). However, these methods typically did not reflect individual differences in spatial dimension of eye movements such as ROI choices (see Le Meur & Bassino, 2013, for a review). von der Malsburg and Vasishth (2011) proposed the Scasim method to quantify similarities among fixation sequences by using an edit distance between fixations in visual angle instead of predefined ROIs. Using the similarities, the fixation sequences are then embedded into a vector space using multi-dimensional scaling (MDS) for further analysis, e.g., K-means clustering. While the similarity measure reflects both spatial and temporal dimension of fixation sequences, the analysis in the vector space may have limited interpretability with respect to the underlying fixation sequences due to the one-way mapping of the MDS embedding (called the “pre-image” problem of similarity-based embedding methods). For example, given a group centroid vector discovered with k-means clustering, a novel fixation sequence representing the group centroid (i.e., the group representative strategy) cannot be recovered due to the one-way mapping of the embedding. Instead, the fixation sequence in the dataset mapped closest to the centroid is used as a ‘prototype’ for the group.

**Fig. 1:**
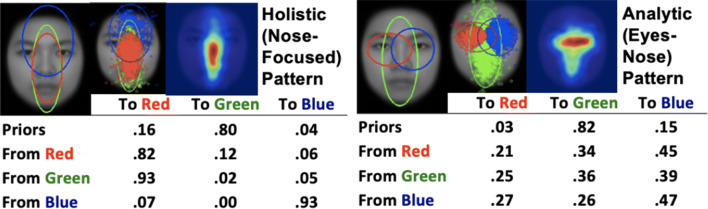
Holistic (nose-focused) and analytic (eyes-nose) eye-movement patterns discovered in face recognition (Chan et al., 2018). *Ellipses* show ROIs as 2-D Gaussian emissions. The table shows transition probabilities among the ROIs. Priors show the probabilities that a fixation sequence starts from an ROI. The two smaller images show the assignment of actual fixations to the ROIs and the corresponding heatmap

Thus, to better understand individual differences in eye-movement patterns, quantitative measures of eye-movement patterns that reflect individual differences in both spatial and temporal dimensions of eye movements are required. Recently there have been attempts of using machine-learning methods to infer characteristics of the observer from eye-movement data (e.g., Kanan et al., 2015). These studies typically use classifiers to discover eye-movement features important for distinguishing two or more observers. However, the classifiers only look for features important for separating the observers, and do not tell us about eye-movement patterns associated with a particular observer. To address this issue, we have recently proposed a novel machine learning-based approach for eye-movement data analysis, eye-movement analysis with hidden Markov models (EMHMM; Chuk et al., 2014; HMM is a type of time-series statistical model in machine learning). EHMMM takes individual differences in spatial (eye fixation locations) and temporal dimensions (the order of eye fixation locations) of eye movements into account (EMHMM Matlab toolbox is available at http://visal.cs.cityu.edu.hk/research/emhmm/). With EMHMM, a sequence of viewed regions of interest (ROIs) is represented by a hidden state sequence, which evolves according to a Markov process where the currently viewed ROI (state) depends on the previously viewed ROI. Since only the fixation locations are observed (measured by the eye tracker), and not their corresponding ROIs, the ROI sequence is hidden and must be inferred from the fixation sequence. Previous studies using HMMs/probabilistic models for modelling eye movement/visual attention and cognitive behavior typically used hidden states of the models to represent cognitive states. For example, Liechty et al. (2003) used two hidden states to represent global and local covert attention in a visual attention model (see also Simola et al., 2008). Yi and Ballard (2009) used states in a dynamics Bayes network to represent subtask goals in modeling task control in sandwich making (see also Hayhoe & Ballard, 2014). Other approaches using Gaussian mixture models (Eckhardt et al., 2013) and heat maps (Caldara & Miellet, 2011) ignore the temporal information in eye movements. In contrast, here we directly use HMMs to model eye fixation sequences (i.e., the inputs of the models are sequences of fixation locations), and hidden states of the models directly correspond to ROIs of eye movements. Each ROI is modelled by a Gaussian emission, which represents the distribution of fixation locations when the individual viewed that ROI^1^. This allows us to discover ROIs and transitions among ROIs specific to an individual (see also Chuk et al., 2020, for EMSHMM, which models transitions of both ROIs and cognitive states using a two-level switching HMM). In addition, to account for individual differences, we use one HMM to model one person’s eye-movement pattern in terms of both person-specific ROIs and transitions among the ROIs. An individual’s HMM is estimated from the individual’s data using a variational Bayesian approach that can automatically determine the number of ROIs. Since HMMs correspond to probability distributions of time-series, the individual HMMs can be clustered according to the similarities of their corresponding probability distributions (using the variational HEM algorithm; Coviello et al., 2014) to reveal representative common patterns, such as the holistic (i.e., mainly looking at the face center) vs. analytic (i.e., looking at both the face center and individual eyes) patterns in face recognition (Fig. 1). Differences among individual eye-movement patterns can be quantitatively assessed using data log-likelihood measures, that is, the log-likelihood of a person’s eye-movement data being generated by the model of a representative common pattern. This log-likelihood measure reflects the similarity between a person’s eye-movement pattern and the representative common pattern, such as how holistic or how analytic a person’s eye-movement pattern is in face recognition. Thus, this method is particularly suitable for examining individual differences in eye-movement pattern and their associations with other cognitive measures. Also, since EMHMM is a Bayesian probabilistic time-series model, it works well with a limited amount of data, in contrast to deep learning models that generally require large amounts of data to train effectively.

We have successfully applied EMHMM to face recognition research, discovering novel findings thus far not revealed by existing methods. For example, we discovered two common eye-movement patterns, holistic pattern vs. analytic pattern, for face learning and recognition through clustering. Since each participant’s eye-movement pattern during face learning or recognition could be classified as a holistic or an analytic pattern according to the clustering results, we found that around 40% of the participants used different patterns between learning and recognition. Participants who used the same or different patterns during learning and recognition did not differ in recognition performance, in contrast to the scan path theory (Noton & Stark, 1971a, 1971b), which posits that eye movements during learning have to be recapitulated during recognition for the recognition to be successful (Chuk et al., 2017a). We also found the analytic eye-movement (eyes-nose) pattern during recognition was associated with better recognition performance as compared with the holistic (nose-focused) pattern (Fig. 1), suggesting that retrieval of diagnostic information (i.e., the eyes) is a better predictor for performance (e.g., Chuk et al., 2017b; Chuk et al., 2017a; Hsiao et al., 2021; Chan et al., 2018). In another study, we found that local attention priming using hierarchical letter stimuli made participants’ eye movements more analytic and increased their recognition performance, as compared with no priming or global attention priming conditions (Cheng et al., 2015). This result suggests an association between engagement of local attention and analytic eye-movement patterns, which consequently improve recognition performance. These findings are consistent with the recent visual recognition literature showing that analytic/featural information is important for recognition in addition to holistic/configural information (Cabeza & Kato, 2000; Cheng et al., 2018; Hsiao & Galmar, 2016; Tso et al., 2020; Tso et al., 2014).

In particular, our recent research using EMHMM has suggested that individuals have preferred eye-movement patterns for face processing that are impervious to the influence of transitory mood changes (An & Hsiao, in press) and able to predict not only recognition performance but also cognitive abilities, particularly executive and visual attention functions (Chan et al., 2018). For example, we found that more older adults adopted the holistic (nose-focused) pattern in face recognition whereas more young adults used the analytic (eyes-nose) pattern, and that eye-movement pattern similarity to the representative holistic pattern predicted lower cognitive status in older adults as assessed using Montreal Cognitive Assessment (HK-MoCA, Yeung et al., 2014). In a second experiment, this correlation was replicated with new participants viewing new face images using the representative HMMs from the first experiment. This finding suggests the possibility of developing representative HMMs from the population for cognitive screening purposes. In a brain imaging study with young adult participants, we found that holistic patterns in face recognition were associated with lower activation in brain regions important for top-down control of visual attention including frontal eye field and intraparietal sulcus (Chan et al., 2016). In another study (Zhang et al., 2019), we found that insomniacs’ impaired ability for facial expression judgments was associated with their use of an eye-movement pattern that focused on the nose and mouth regions but not the eyes, suggesting impaired visual attention control (see also Chan et al., 2020a, b). In tasks other than face processing, we found that a more centralized eye-movement pattern when viewing documentary videos was associated with better comprehension and better executive function ability (as assessed using a problem-solving task, Tower of London. Zheng et al., 2019). Together these results suggest the possibility of using eye tracking as an easily deployable screening assessment for cognitive deficits.

While we have been successful in using the EMHMM method to understand individual differences in eye movements in some visual tasks, particularly in face recognition, currently it is limited to tasks involving stimuli with the same feature layout (e.g., faces) so that discovered ROIs correspond to the same features across stimuli (e.g., individual facial features). Nevertheless, in many real-life tasks, such as scene perception, website browsing, or reading, stimuli have different feature layouts. Thus, looking at the same location across stimuli does not usually correspond to looking at the same feature, and eye fixations on similar features across stimuli do not usually correspond to looking at the same location. As the EMHMM approach summarizes participants’ eye-movement patterns based on only fixation locations, it does not have the capacity to discover consistent perceptual styles across stimuli with different feature layouts (such as the preference to mainly look at the foreground or the background) either within a participant or across participants. There is also no existing eye-movement data analysis method that can summarize and compare individuals’ perceptual styles across stimuli with different feature layouts from both the temporal and spatial dimensions of eye movements. In order to replicate our success in research fields other than face recognition and have a broader impact on cognitive research using eye tracking, it is essential to develop new EMHMM methodologies for these scenarios.

**Fig. 2:**
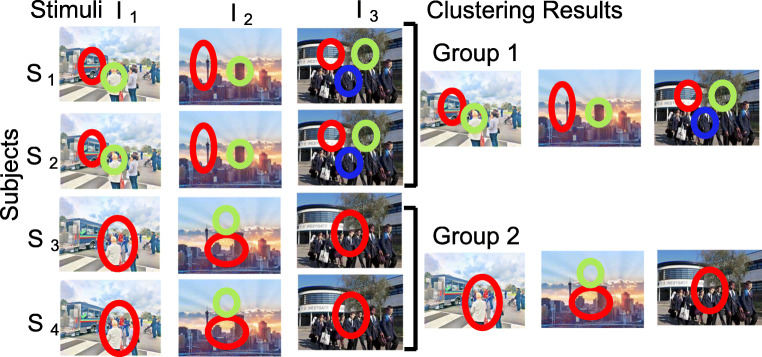
Illustration of EMHMM with co-clustering. *Left*: each subject (S_i_) has an HMM to summarize the eye-movement pattern for each stimulus (I_i_). *Circles* indicate ROIs. *Right*: The co-clustering algorithm groups together participants whose eye-movement patterns were similar to one another when viewing each of the stimuli (I_i_) consistently. In this example, S_1_ and S_2_ are clustered together to form Group 1 since their eye-movement patterns were similar to each other when viewing I_1_, I_2_, and I_3_ consistently, and a representative HMM is generated for each stimulus (I_i_) by summarizing the eye-movement patterns of the stimulus from S_1_ and S_2_ using an HMM; similarly for S_3_ and S_4_ forming Group 2

We have previously proposed inclusion of perceived images as features in the ROI representations. While this method has significantly improved discovery of ROIs (Brueggemann et al., 2016), it does not work well when features among stimuli differ significantly, such as in scene viewing. Here we propose a new method: we model each participant’s eye-movement pattern when viewing a particular stimulus with one HMM, and then use the data mining technique co-clustering (e.g., Govaert & Nadif, 2013) to discover participants sharing similar eye-movement patterns across stimuli. The co-clustering formulation ensures that the participant groups are consistent across all stimuli. Thus, this method is particularly suitable for discovering representative perceptual styles in a visual task among participants under the assumption that each participant exhibits a consistent perceptual style across the stimuli^2^. The result is a grouping of participants and their representative HMMs for each stimulus (Fig. 2). The similarity between an individual’s eye-movement patterns across stimuli and the representative HMMs of a participant group can be quantitatively assessed using log-likelihood measures as in the existing approach, or more specifically, by summing over the log-likelihood measures of the individual’s eye movements across the representative HMMs (see the Data Analysis section for details). This similarity measure then can be used to examine the relationship between eye-movement patterns and other cognitive measures. In addition, we use a variational Bayesian approach to automatically determine the optimal number of participant groups (following a similar approach to Lan et al., submitted). We provide the programs for performing this analysis method as a Matlab toolbox, EMHMM with Co-clustering, publicly available to the community under an open-source license agreement.

To demonstrate the advantage of the EMHMM with co-clustering method in understanding individual differences in eye-movement pattern in visual tasks that involve stimuli with different feature layouts, here we use scene perception as an example, since visual scenes differ significantly in feature layout and characteristics, providing a challenging scenario for the new methodology. Recent research has shown that scene perception involves complex and dynamic perceptual and cognitive processes that can be influenced by both observers’ goals and diverse scene properties at multiple levels (Malcolm et al., 2016). Eye movements during scene perception have been found to reflect the complexity of cognitive processes involved, and thus can potentially provide rich information about individual differences in perception styles and cognitive abilities. Here we aimed to use EMHMM with co-clustering to examine individual differences in eye-movement pattern and their associations with object recognition performance and cognitive abilities during scene viewing. We use scene images from both natural and man-made/urban environments to increase stimulus generality, as they have significantly different global scene properties that could be distinguished by humans the most efficiently (Greene & Oliva, 2009; Joubert et al., 2007). Specifically, we examined: (1) what types of scene images (animals in nature vs. vehicles in a city) could induce larger individual differences in eye-movement pattern, and (2) whether eye-movement patterns during scene viewing are associated with subsequent foreground object recognition performance and cognitive abilities. Previous studies have reported two eye-movement patterns in scene perception: one looking mainly at the foreground object, whereas the other looking at both the foreground and the background and switching between them; also, the latter pattern was associated with better performance in foreground object recognition with the old background (Chua et al., 2005). Accordingly, we predicted to discover similar eye-movement patterns in our participants, and individual differences in eye-movement pattern would be associated with foreground object recognition performance. In addition, our previous studies (e.g., Chan et al., 2018; Zheng et al., 2020) have shown that eye-movement patterns in visual tasks are associated with cognitive abilities, particularly in executive function, visual attention, and working memory. We predicted that eye movements in scene perception would also be associated with these abilities. Also, as previous research has suggested category-specific attention to animals (New et al., 2007), animals with a natural background may catch more initial attention and subsequently better reveal individual differences in analytic/holistic style than scenes with man-made objects.

## Methods

### Participants

In the examination on face recognition performance difference between participants using different eye-movement patterns (i.e., analytic vs. holistic), Chuk et al. (2017b) observed a large effect size. A power analysis showed that 52 participants were required to observe a group difference with a large effect size (d = 0.8), α = 0.5, and power = 0.8. Accordingly, we aimed to recruit 60 Asian participants. In the end, 61 participants participated (as one participant cancelled the booking but still showed up for the experiment). They were 35 females and 26 males, aged 18–25 (M = 20.77, SD = 1.70), from the University of Hong Kong. All participants had normal or corrected-to-normal vision.

### Materials

The materials consisted of 150 scene images with animals in a natural environment and 150 scene images with vehicles in an urban environment from the Internet. Images with different numbers of foreground objects, feature layouts, and locations of foreground objects were used to increase stimulus variability to provide adequate opportunities to elicit individual differences in eye-movement pattern. All images were adjusted to equalize brightness and contrast.

### Design and procedure

The scene perception task consisted of a passive viewing phase and a surprise recognition phase (following Chua et al., 2005). During the passive viewing phase, for each scene type (animals vs. vehicles), participants were presented with 60 images one at a time, each for 5 s, and rated from 1 to 5 how much they liked the image. During the surprise recognition phase, for each scene type, participants were presented with 60 images with old foreground objects, with half of them presented in the same old background and the other half in a new background, together with the same number of lure images with new objects in a new background. They were presented with the images one at a time and judged whether they saw the foreground object during the passive viewing phase. The image stayed on the screen until response. In both phases, the animal and vehicle scene images were presented in two separate blocks, with the block order counterbalanced across participants. This block design was chosen in order to reduce the influence from switching between different image types during the experiment in examining possible eye-movement pattern difference between viewing animal and vehicle images. Participants’ eye movements were recorded using an EyeLink 1000 eye tracker, with a chinrest to minimize head movements. Each trial started with a fixation cross at the screen center. The experimenter initiated the image presentation when a stable fixation was observed at the fixation cross. An image was then presented at the screen center, spanning 35° x 27° of visual angle at a viewing distance of 60 cm. Before each block, a nine-point calibration procedure was performed. Re-calibration took place whenever drift correction error exceeded 1° of visual angle.

In addition, participants performed three cognitive tasks to examine whether their eye-movement patterns were associated with cognitive abilities.

1. Tower of London task (Phillips et al., 2001) for testing executive function/planning abilities: In each trial, participants saw three beads randomly placed on three pegs as the starting position, together with the target position. They were asked to move one bead at a time to reach the target position as quickly as possible with the minimum number of moves and to plan the moves in mind before execution. In total there were ten trials. The total number of extra moves, number of correct trials, total planning time before executing the first move, and total execution time for the moves were measured.
2. Flanker task (Ridderinkhof et al., 1999) for testing selective attention: Participants judged the direction of an arrow flanked by four other arrows. In the congruent condition, the flanking arrows pointed in the same direction as the target arrow, whereas in the incongruent condition, the flanking arrows pointed at the opposite direction. In the neutral condition, the flankers were non-directional symbols. In total there were 120 trials, with 40 trials in each condition.
3. Verbal and visuospatial two-back task for assessing working memory capacity (Lau et al., 2010): In each trial, participants judged whether the presented symbol/symbol location was the same as the one presented two trials back in the verbal/visuospatial task, respectively. There were 50 trials in each of the verbal and visuospatial two-back tasks.

### Data analysis

We analyzed participants’ eye movements during the passive viewing phase using EMHMM with co-clustering. First, each participant’s eye movements for viewing each stimulus were summarized using an HMM. In particular, an individual’s HMM was estimated from a participant’s eye-movement data using a variational Bayesian approach, where prior distributions are placed on the HMM parameters (e.g., transition matrix, Gaussian emissions, prior probabilities), and a posterior distribution of the individual’s HMM is estimated given the data. The optimal hyperparameters of the prior distributions and number of ROIs are determined by maximizing the *marginal* log-likelihood of the data, which automatically trades off between model fit and model complexity.^3^ To determine the number of ROIs for an individual’s HMM, we learn HMMs with different numbers of ROIs (within the preset range of 2–4, inclusive) via optimizing the hyperparameters. We then select the resulting HMM with the highest marginal log-likelihood. Second, individual HMMs for viewing each stimulus were clustered to discover representative patterns among the participants. Co-clustering was used to cluster participants into groups according to whether they used similar eye-movement patterns across the stimuli (Fig. 2). In particular, the variational Bayesian hierarchical EM (VBHEM) algorithm (Lan et al., submitted), which clusters a set of individuals’ HMMs for viewing a stimulus into groups using the variational Bayesian approach to determine the optimal number of groups and forms representative HMMs for each group, was modified to perform co-clustering over several sets of HMMs, where each set corresponds to individuals’ HMMs for one stimulus. VBHEM assumes prior distributions on the parameters of the representative HMMs and their cluster probabilities and aims to find representative HMMs that have maximum expected marginal log-likelihood according to time-series distributions of the individuals’ HMMs. The optimal hyperparameters of the prior distributions and number of clusters is automatically determined by maximizing the expected *marginal* log-likelihood of the data. We did not place a prior on the number of clusters, since the Dirichlet prior on the cluster probabilities already naturally selects the number of clusters. To obtain the optimal number of clusters, we learn representative HMMs for different number of clusters (from 1 to 5, inclusive) by optimizing the hyperparameters. We then select the resulting model (number of clusters) with highest expected marginal log-likelihood. VBHEM with co-clustering is equivalent to running VBHEM separately on each set of HMMs (one set for each stimulus), except that it computes consistent cluster assignments of individuals to groups across all stimuli (i.e., all runs of VBHEM) such that a participant would be consistently assigned to the same group across all sets of HMMs. The result of co-clustering is a set of representative HMMs (one for each stimulus) for each group.

A formal derivation of VBHEM with co-clustering can be found in the Appendix. Note that we have previous extended the variational hierarchical EM (VHEM) algorithm (Coviello et al., 2014) to perform co-clustering (Hsiao et al., 2019), which clusters a set of individuals’ HMMs into groups and forms representative HMMs for each group across each stimulus, using a prespecified number of clusters. In the Appendix we also provided a formal derivation of VHEM with co-clustering. Using VHEM with co-clustering, instead of VBHEM with co-clustering, is provided as an option in the EMHMM with Co-clustering toolbox. Note that in both versions of co-clustering, the number of ROIs used for generating the representative HMMs for each stimulus can be determined separately. Thus, the representative HMMs of different stimuli can have different numbers of ROIs. In the current version of the EMHMM with co-clustering toolbox, the default setting is to set the number of ROIs of the representative HMMs for each stimulus to be the median number of ROIs of the individual HMMs,^4^ and the number of ROIs in an individual HMM is determined using a variational Bayesian approach to derive the optimal number of ROIs from a pre-set range (here we set it to be from 2 to 4 ROIs) given the data (thus, individual HMMs may have different numbers of ROIs). However, the number of ROIs in both individual HMMs and representative HMMs can be changed according to the user’s analysis requirements. Also, the toolbox will remove an ROI (or in other words, a hidden state) that is not used after training in both individual and representative HMMs. Here we reported the results using VBHEM with co-clustering and the default setting.

To examine whether animal (natural) or vehicle (man-made) images could induce larger individual differences in eye-movement pattern, we quantified the difference between any two representative patterns for each stimulus as the result of co-clustering via the symmetric KL (SKL) divergence between the two group HMMs. The SKL is given by (KL_12_+KL_21_)/2, where KL_12_ is the KL divergence between the probability distribution of fixation sequences from the Group 1 representative HMM and that of Group 2, and vice-versa for KL_21_ (KL is not symmetric). More specifically, KL_12_ is estimated by the log-likelihood of Group 1 data (fixation sequences) being generated by Group 1 HMM minus the log-likelihood of Group 1 data being generated by Group 2 HMM (Chuk et al., 2014). Similarly, KL_21_ is estimated by the log-likelihood of Group 2 data being generated by Group 2 HMM minus the log-likelihood of Group 2 data being generated by Group 1 HMM. Using SKL to quantify the difference between representative patterns thus takes both fixation locations and the temporal order the fixations into account. We then compared SKL measures for animal and vehicle images and examined the characteristics of the images that typically led to larger SKL.

We also examined whether the two groups of participants as the result of co-clustering differed in performance in foreground object recognition and the cognitive tasks. To examine the correlations between eye-movement pattern and recognition performance/cognitive abilities, we quantified the similarity of a participant’s eye-movement pattern to the group patterns using a cluster score, CS = (L_1_-L_2_) / (|L_1_|+|L_2_|), where L_1_ and L_2_ are the log-likelihoods of a participant’s data being generated under Group 1 and Group 2 pattern, respectively (Chan et al., 2018). Larger/positive values of CS indicate higher similarity to Group 1, and smaller/negative values indicate similarity to Group 2.

## Results

### Eye-movement patterns in scene perception

On average, participants made 13.42 fixations per trial (SD = 1.81) when they passively viewed scene images, each for 5 s. Using the eye-movement data from 61 participants on 120 image stimuli, EMHMM with co-clustering discovered two groups from the data. Group 1 contained 33 participants and Group 2 contained 28 participants. The co-clustering model estimates two representative HMMs for each image stimulus, corresponding to the representative patterns of Groups 1 and 2. Clustering algorithms may sometimes find overlapped clusters, when there is not a large separation in the data. Thus, to examine whether participants in the two groups as the result of co-clustering had statistically significantly different eye-movement patterns, for each participant we calculated Group 1 data likelihood as the log-likelihood of the participant’s eye-movement data being generated from the Group 1 representative HMMs (averaged across the HMMs of the stimuli), and similarly for Group 2 data likelihood. If participants in Group 1 and Group 2 had significantly different eye-movement patterns, we expected that participants in Group 1 should have larger Group 1 data likelihood than Group 2 data likelihood, whereas those in Group 2 should have larger Group 2 data likelihood than Group 1 data likelihood (Chuk et al., 2014). The results showed that participants in Group 1 had significantly higher Group 1 data likelihood than Group 2 data likelihood, *t*(32) = 15.40; *p* < 0.001, *d* = 2.68, 95% CI [0.22, 0.28]. Similarly, participants in Group 2 had significantly higher Group 2 data likelihood than Group 1 data likelihood, *t*(27) = 8.96; *p* < 0.001, *d* = 1.69, 95% CI [0.18, 0.28]. This result demonstrated that participants in Group 1 and Group 2 had significantly different eye-movement patterns in scene perception.

**Fig. 3:**
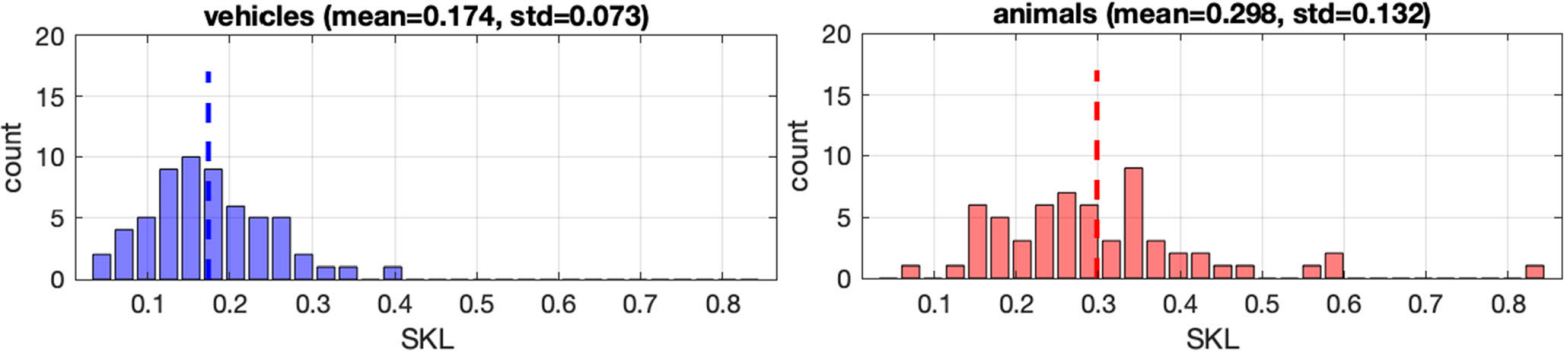
Histograms of SKL divergence between Group 1 and Group 2 patterns on vehicle or animal images. In general, animal images induced larger SKL divergence between the two groups than vehicle images

We also examined which types of images, animal or vehicle images, induced larger eye-movement pattern difference between participants in Group 1 and Group 2. For each image, the difference between the representative patterns in Group 1 and Group 2 was measured in SKL. As shown in Fig. 3, animal images induced larger differences in eye-movement pattern between Group 1 and Group 2 than vehicle images, *t*(118) = – 6.39; *p* < 0.001, *d* = – 1.17, 95% CI [– 0.33, – 0.17].

**Fig. 4:**
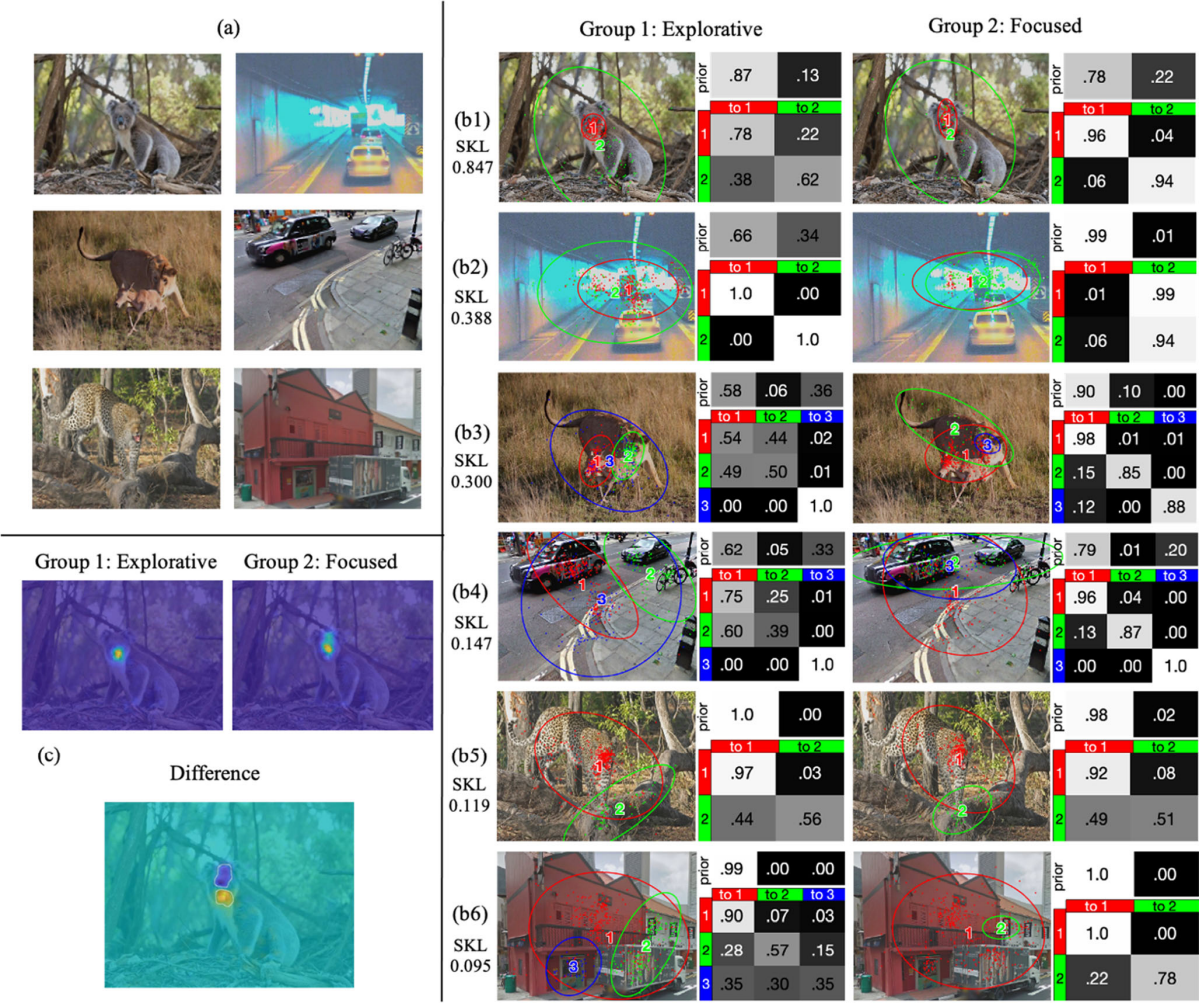
**a** Example stimuli. **b** Representative HMMs of the example stimuli in the two participant groups resulting from co-clustering. The two groups showed larger difference in eye-movement pattern when viewing the first three images and smaller difference when viewing the last three images, as measured by symmetric KL divergence (SKL). **c** Heatmap plots of eye fixations on the first image for the two groups, and the difference regions (*orange* for Group 1, *blue* for Group 2). The explorative group looked at the center of the koala’s face more whereas the focused group looked at the eye region of the koala’s face more

**Fig. 5:**
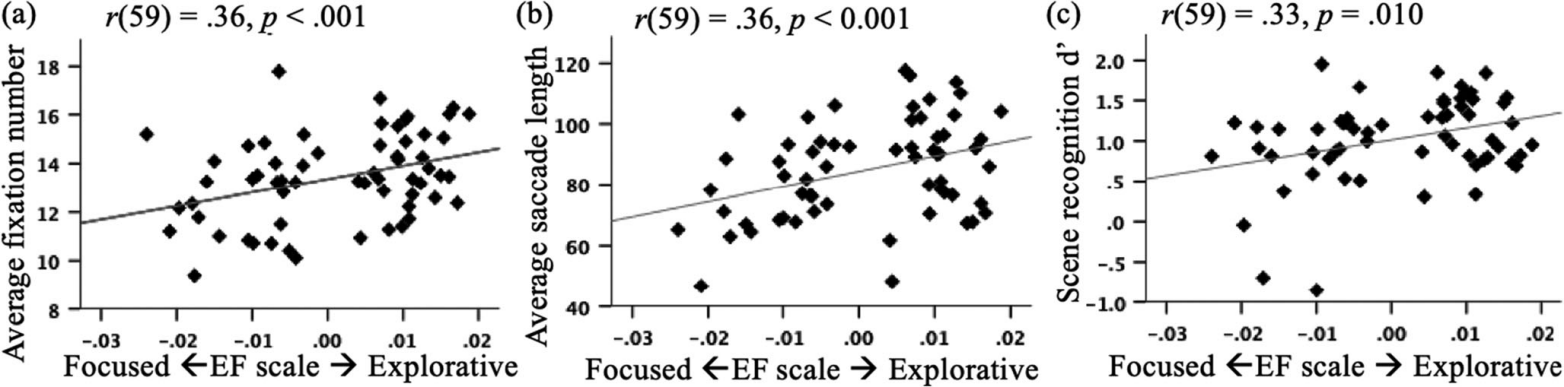
Correlation analysis between EF Scale and **a** average number of fixations, **b** average saccade length, and **c** scene recognition performance in d’

Figure 4 presents six example images and their corresponding representative HMMs in Group 1 and Group 2. Figure 4b1 to 4b3 shows two example images where the corresponding representative HMMs for the two groups had a large SKL difference. As can be seen in the figure, Group 2 focused more on the foreground object and showed a higher probability to stay looking within the same ROI, while Group 1 explored the image more by looking at both the foreground object and the background and exhibited higher probabilities to transit among different ROIs. When looking at an animal face, Group 2 focused more on the eyes, whereas Group 1 looked at the nose (Fig. 4c). Figure Fig. 4b4 to Fig. 4cd6 shows example images where participants in the two groups had similar eye-movement patterns. In general, larger differences in eye-movement pattern between the two groups occurred on images where the foreground object (animal or car) was salient as compared with the background, e.g., an animal among trees, or a car on a road. As humans have category-specific attention for animals (e.g., New et al., 2007), which make animals more salient than vehicles, the animal images generally induced larger individual differences in eye-movement patterns than vehicle images. In contrast, images with cluttered backgrounds and non-interesting foreground objects typically induced similar eye-movement patterns. For current purposes, we referred to Group 1 and 2 patterns as the explorative and focused pattern, respectively, and the cluster score between the two group patterns as the explorative-focused (EF) scale. Indeed, participants using the explorative pattern had a larger average number of fixations, *t*(59) = 2.45, *p* = < .017, *d* = 0.63, 95% CI [0.20, 1.99], and longer saccade lengths, *t*(59) = 2.33, *p* < .023, *d* = 0.60, 95% CI [1.31, 17.33], than those using the focused pattern, and the more similar their eye-movement pattern to the explorative pattern (in EF scale), the larger the average number of fixations, *r*(59) = 0.358, *p* < .001, 95% CI [0.12, 0.56], and saccade length, *r*(59) = 0.362, *p* < .001, 95% CI [0.12, 0.56] (Fig. 5a and b). As EMHMM does not use sequence length or saccade length information, the differences in average number of fixations and saccade length emerged naturally as the result of the clustering. These results were consistent with the interpretation that Group 1 was more explorative than Group 2.

### Reliability of the log-likelihood measures in assessing one’s eye-movement pattern

To examine the reliability of the log-likelihood measures and the EF scale in assessing one’s eye-movement pattern in scene perception, we performed split-half reliability test (Spearman–Brown corrected) by splitting the 60 images into two halves of 15 animal and 15 vehicle images each. The results showed that both the log-likelihood measures (log-likelihood measure of the explorative pattern, r_sb_ = .96; log-likelihood measure of the focused pattern, r_sb_ = .98) and the EF scale (r_sb_ = .99) had excellent split-half reliability. This result suggested that individual participants exhibited consistent eye-movement patterns across the scene images as assessed using the EMHMM method. Note that in the current study, the discrimination sensitivity d’ in the scene recognition task (120 trials in total) had good split-half reliability (r_sb_ = .88) and average reaction time had excellent split-half reliability (r_sb_ = .99). This result suggested that reliability of the eye-movement pattern measure using EMHMM was comparable to and sometimes exceeded that of performance measures.

In a separate analysis, we examined the consistency of individual participants’ eye-movement pattern in viewing animal vs. vehicle images. The results showed that when we performed split-half reliability test by splitting the images according to image type (animal vs. vehicle), both the log-likelihood measures (log-likelihood measure of the explorative pattern, r_sb_ = .80; log-likelihood measure of the focused pattern, r_sb_ = .87) and the EF scale (r_sb_ = .92) still had good to excellent reliability. This result suggested that individuals had consistent eye-movement patterns across image types as assessed using EMHMM.

### Does eye-movement pattern predict foreground object recognition performance?

To examine whether participants using explorative and focused eye-movement patterns differ in foreground object recognition performance, a 2 x 2 ANOVA on recognition performance was conducted with eye-movement group (Group 1 explorative pattern vs. Group 2 focused pattern) as a between-subject variable and image background (old vs. new) as a within-subject variable. The results showed a main effect of group, *F*(1, 59) = 5.79, *p* = .019, η^2^ = 0.089: participants using the explorative pattern performed significantly better than those using the focused pattern. This effect interacted with image background, *F*(1, 59) = 4.54, *p* = .037, η^2^ = 0.072: the advantage of the explorative pattern in foreground object recognition performance was significantly larger when the foreground object was presented with the old background than with a new background, although the advantage was significant in both cases (old background: *t*(59) = 2.41, *p* = .019, *d* = 0.62, 95% CI [0.08, 0.85]; new background: *t*(59) = 2.15, *p* = .036, *d* = 0.55, 95% CI [0.01, 0.38]). Consistent with these findings, correlation analysis showed that participants’ eye-movement pattern similarity to the explorative pattern (EF scale) was correlated positively with scene recognition performance in d’, *r*(59) = .326, *p* = .010, 95% CI [.081, .534] (Fig. 5c). These findings suggest that the explorative strategy was associated with better foreground object recognition performance.

To understand what factors best accounted for participants’ foreground object recognition performance, we performed a stepwise multiple regression analysis predicting recognition performance with all cognitive ability measures in addition to EF scale. The results showed that only EF scale, β = .331, *p* = .012, and visuospatial two-back task RT, β = .308, *p* = .012, were significant predictors, *R*^*2*^ = .171, *F*(2, 57) = 7.090, *p* = .002. The tests for multicollinearity indicated a low level of multicollinearity for both EF scale (tolerance = .999) and visuospatial two-back task RT (tolerance = .999). This result suggested that a linear combination of participants’ visuospatial working memory ability and online eye-movement pattern during scene perception best predicted their foreground object recognition performance. In a hierarchical multiple regression analysis predicting foreground object recognition performance, with visual spatial two-back task RT entered at the first step, we found that EF scale explained 10.3% additional variance and the change in *R*^*2*^ was significant, *p* = .009. This finding suggested that after working memory ability was controlled, a person’s online eye-movement pattern still contributes significantly to foreground object recognition performance in scene perception.

### What cognitive abilities predict eye-movement pattern?

To examine what cognitive abilities best accounted for individual differences in eye-movement pattern, we performed a stepwise multiple regression analysis predicting EF scale with all cognitive ability measures. The results showed that flanker task accuracy in congruent trials, β = – .300, *p* = .020, contributed significantly to the regression model, *R*^*2*^ = .074, *F*(1,58) = 5.719, *p* = .020: the more explorative the pattern, the lower the flanker task accuracy. There was a low level of multicollinearity for both flanker task accuracy in congruent trials (tolerance = 1.000). This result suggested that eye-movement pattern in scene perception is particularly related to visual attention.

## Discussion

Here we proposed a new methodology for summarizing and quantitatively assessing individual differences in eye-movement pattern in tasks involving stimuli with different feature layouts by combining the EMHMM approach (Chuk et al., 2014) with the data mining technique co-clustering. We used one HMM to summarize a participant’s eye-movement pattern when viewing a particular stimulus, and then used co-clustering to discover participants sharing similar eye-movement patterns across stimuli. By applying this method to young Asian adults’ eye movements during scene perception, we discovered explorative (switching between the foreground object and the background) and focused (looking mainly at the foreground object) eye-movement patterns. These patterns were similar to those observed in Asians and Caucasians respectively in previous studies (e.g., Chua et al., 2005). Note, however, that our participants were all Asian, suggesting substantial individual differences in eye-movement pattern during scene viewing even within a culture. Interestingly, for images containing animal faces, participants who focused more on the foreground object (i.e., the focused eye-movement pattern) looked more to the eyes of the animal faces, suggesting engagement of local attention (Miellet et al., 2011). In contrast, those who switched between the foreground object and the background more often (i.e., the explorative pattern) looked more to the animal face center, suggesting engagement of global processing. This result is consistent with the literature reporting that Caucasians focused more on the foreground object in scene perception and the eyes in face recognition, suggesting more engagement in local attention, whereas Asians’ looked more often at the background in scene perception and the face center in face recognition, suggesting more engagement in global attention (e.g., Blais et al., 2008). This result also suggests that one’s eye-movement pattern in scene perception may be associated with that in face recognition, or in other words, individuals may have a consistent perception style across different stimulus types (e.g., Rayner et al., 2007). Future work will examine this possibility. Note also that using EMHMM, Chuk et al. (2017b) showed that Asians and Caucasians did not differ in the frequency of adopting the analytic (eyes-nose) or holistic (nose-focused) eye-movement patterns, suggesting little modulation from culture on eye-movement pattern when individual difference was taken into account. Future work will examine whether a similar phenomenon can also be observed in scene perception through EMHMM with co-clustering.

We also observed that participants adopting the explorative eye-movement pattern had better foreground object recognition performance than those using the focused pattern regardless of whether the foreground object appeared in a new or old background, although this advantage was larger when the foreground object appeared in the old than a new background. This finding was generally consistent with Chua et al. (2005), in which Asians, who looked more often at the background than Caucasians, performed better in foreground object recognition with the old background. Our finding suggests that a more explorative eye-movement pattern during scene perception may be beneficial for remembering the foreground object due to more retrieval cues available through exploration. Consistent with this speculation, it has been shown that associative processing is inherent in scene perception (Aminoff & Tarr, 2015), suggesting that an explorative eye-movement pattern may facilitate associative processing and consequently enhance scene memory. This finding is in contrast to face recognition literature, where the analytic or more eye-focused eye-movement pattern (which is associated with engagement of local attention and the focused eye-movement pattern reported here) was reported to lead to better recognition performance due to better retrieval of diagnostic features, the eyes (Chuk et al., 2017b). Our multiple regression analysis results further showed that participants’ foreground object recognition performance depended on both their visuospatial working memory ability and eye-movement pattern during scene perception. More specifically, a person’s online eye-movement pattern during scene perception contributes significantly to foreground object recognition performance even after one’s cognitive (working memory) ability is accounted for. This result provides evidence suggesting that one’s memory of objects in a scene may be improved with instructions on the use of an optimal eye-movement pattern.

While the explorative eye-movement pattern was associated better foreground object recognition performance, our multiple regression analyses also showed that the focused eye-movement pattern was associated with better performance in the congruent trials of the flanker task, suggesting better abilities in visual attention. The advantage of the focused pattern in the congruent but not incongruent or neutral trials in the flanker task suggested that participants adopting this pattern might have better feature integration abilities. This finding was generally consistent with previous research: as participants adopting the focused pattern preferred to look at the eyes of the animals, the focused pattern may be related to the analytic/more eyes-focused eye-movement pattern in face recognition, which was associated with better face recognition performance, visual attention (the trail making task), and executive function (the tower of London task; Chan et al., 2018). Future work will examine whether the focused pattern in scene perception is indeed associated with the analytic/eyes-nose pattern in face recognition (Fig. 1).

We also observed that images with a salient foreground object relative to the background tended to induce large individual differences in eye-movement pattern, and that animal images induced larger individual differences than vehicle images (Fig. 4). This phenomenon may be due to our category specific attention to animals (New et al., 2007) that made them more salient than vehicles or other object types, providing better opportunities to induce the difference between the explorative and focused eye-movement patterns. It could also be a reflection of some fundamental differences between animal and vehicle images such as the characteristics of the backgrounds. This finding has important implications for the possibility of using eye tracking to provide screening tools for cognitive disorders (Chan et al., 2018), as images that induce larger individual differences will be more likely to provide adequate variance among individuals for identifying atypically eye-movement patterns.

In contrast to the original EMHMM method, which is limited to visual tasks involving stimuli with the same feature layouts such as faces, EMHMM with co-clustering can be applied to a large variety of visual tasks that involve stimuli with different feature layouts, including scene perception, reading, website browsing, video games, etc. It can also be used to summarize a person’s eye-movement pattern across tasks to obtain a personal eye-movement pattern profile. Such a personal profile can be useful for mental health or education-related purposes. For example, using the original EMHMM method, we have previously shown that older adults’ eye-movement patterns during face recognition are predictive of their executive function and visual attention ability (Chan et al., 2018), that impaired facial emotion recognition in individuals with sleep loss is associated with a nose-mouth eye-movement pattern that misses the eye region (Zhang et al., 2019), that social anxiety symptoms are associated with less flexibility in eye-movement strategy when viewing emotional faces (Chan et al., 2020a), and that individuals who view faces with more nose-focused eye-movement patterns interpret illness-related scenarios more negatively (Chan et al., 2020b). With eye movements in a variety of visual tasks summarized using EMHMM with co-clustering, particularly those with real life significance such as reading, website browsing, information system usage, and perception of emotional scenes/faces, we will have a more complete eye-movement pattern profile for each individual to increase the accuracy in early screening of cognitive or socioemotional disorders such as ageing-related cognitive decline or social anxiety, as well as to have a better understanding of the mechanisms underlying these disorders. Similarly, in research on education, we have used EMHMM to show that participants have better comprehension when using a more centralized eye-movement pattern in viewing documentary videos (Zheng et al., 2019). EMHMM with co-clustering will enable us to examine eye movements in a large variety of learning tasks to understand the relationship between eye-movement patterns and learning performance and to discover better eye-movement strategies for learning. A personal eye-movement pattern profile can also be used for predicting a person’s eye-movement behavior under existing or novel situations. For example, a participant’s eye movements for a stimulus that has not been presented to the participant can be inferred from how the participant’s eye movements for other stimuli deviate from the representative HMMs (using the log-likelihood measures). This personal profile has a variety of applications such as video coding, foveated rendering for virtual reality, advertisement, or human computer interaction.

Another possible application of EMHMM with Co-clustering is in the research on cultural differences in perception style. More specifically, it has been reported that Westerners are more likely to attribute the cause of an event to isolated objects (analytic cognition), whereas Asians are more likely to attribute the cause of an event to the context of the event (holistic cognition; Nisbett & Miyamoto, 2005; Ito, Masuda, & Li, 2013; Li et al., in press), and this cultural difference has been argued to be the mechanism underlying eye-movement pattern difference between Asians and Caucasians observed in scene perception (e.g., Chua et al., 2005) and face recognition (e.g., Blais et al., 2008). Nevertheless, this cultural difference has not been consistently reported in the literature. For example, Evans et al. (2009) found no difference between American and Chinese participants in number of fixations and fixation duration to the foreground and background (see also Rayner et al., 2007). Using EMHMM, Chuk et al. (2017b) found no significant difference in eye-movement pattern between Asians and Caucasians in face recognition. EMHMM with Co-clustering will enable researchers to examine this cultural difference across a larger variety of visual tasks.

Finally, we note that the HMMs used in EMHMM assume Gaussian distributions as the emission densities for the ROIs. As 95% of the Gaussian density is within 2 standard deviations of the mean, the Gaussian is a suitable model for an ROI over a compact spatial region in the image. In the case that the actual fixation distribution is non-Gaussian, e.g., a fat-tail distribution, then the HMM will represent it with multiple Gaussian ROIs. For example, for a fat-tail distribution, the HMM can model the “central” and “fat” portions with two Gaussian ROIs with small and large standard deviation, respectively. Future work can consider extending our EMHMM framework to use other distributional assumptions, e.g., fat-tail distributions, for the ROIs, in order to directly represent such distributions if required.

In conclusion, we show that EMHMM with co-clustering can effectively summarize and quantitatively assess individual differences in eye-movement pattern in tasks involving stimuli with different feature layouts, and in turn lead to new discoveries not yet found by existing methods. By applying this to scene perception, we discovered the explorative and focused eye-movement patterns among Asians. Whereas the explorative pattern was associated with better foreground object recognition performance, the focused pattern was associated with better feature integration and planning abilities. Also, images with a salient foreground object relative to the background induced larger individual differences in eye-movement pattern. These discoveries have important clinical and educational implications for the use of eye tracking in cognitive deficit detection and cognitive performance monitoring. Thus, EMHMM with co-clustering can be applied to a large variety of visual tasks and applications, making lasting impacts on how researchers across disciplines use eye movements to understand cognitive behavior.

## Supplementary Information

ESM 1 (PDF 294 kb)

## Footnotes

1 In the EMHMM method, we can add fixation duration as an additional input dimension such that each input data point is represented as fixation location x, fixation location y, and fixation duration. In this case, each hidden state of the HMM corresponds to an ROI associated with a fixation duration distribution. This has been implemented in Chuk et al. (2017a).

2 When participants do not have a consistent perceptual style across the stimuli, the co-clustering will be driven by the consistency exhibited by the majority of the stimuli. Consequently, the representative patterns discovered for a stimulus inconsistent with the majority will tend to be more similar to each other.

3 This method for model selection is also called “empirical Bayes” in statistics or “evidence approximation” in machine learning. See Chapter 5 of Rasmussen and Williams (2006) or Chapter 3.4 of Bishop (2006) for detailed discussions.

4 In principle, the numbers of ROIs for a representative HMM for a stimulus can be estimated automatically by maximizing the expected marginal log-likelihood. However, this requires a search over an exponential number of combinations of numbers of ROIs, which is computationally infeasible. Thus, for simplicity and computational efficiency, we assume the number of ROIs of a representative HMM (for a stimulus) is the median of number of ROIs in the individual HMMs.
