# Supplementary material for: Eye movement analysis with hidden Markov models (EMHMM) with co-clustering
Source: Behav Res Methods. 2021 Apr 30;53(6):2473–86. doi: 10.3758/s13428-021-01541-5 (PMC8613150; doi:10.3758/s13428-021-01541-5)
Supplement: Supplementary file 1 — (PDF 294 kb) [file 13428_2021_1541_MOESM1_ESM.pdf]

Eye Movement analysis with Hidden Markov Models (EMHMM) with co-clustering: Appendix

Janet H. Hsiao
Department of Psychology, University of Hong Kong
The State Key Laboratory of Brain and Cognitive Sciences, University of Hong Kong

Hui Lan
Department of Computer Science, City University of Hong Kong

Yueyuan Zheng
Department of Psychology, University of Hong Kong

Antoni B. Chan
Department of Computer Science, City University of Hong Kong

A. Derivation of VHEM with Co-clustering

In this Appendix we provide the formal derivation of VHEM with co-clustering. The original variational hierarchical EM (VHEM) algorithm (Coviello et al., 2014) clusters a set of individuals' HMMs into groups and forms a representative HMM for each group. Here we modify VHEM to perform co-clustering over several sets of HMMs, where each set corresponds to individuals' HMMs for one stimulus. In particular, VHEM with co-clustering is equivalent to running VHEM separately on each set of HMMs (i.e., for each stimulus), but it computes consistent cluster assignments of individuals to groups across all stimuli (i.e., all runs of VHEM). The output result of co-clustering is a set of representative HMMs (one for each stimulus) for each group.

A.1 Problem formulation

Consider an experiment where subjects view multiple image stimuli, which have different spatial arrangements (e.g., images of different scenes). The goal is to cluster the subjects into groups so that group members share similar eye gaze behaviors across all stimuli, although the shared behavior for a specific stimuli might be different from other stimuli.

Let $\mathcal{B}_i^{(s)}$ be the individual (base) HMM learned for subject i on stimulus s. Table 1 shows the individual HMMs. The goal of co-clustering is to find subsets of rows in Table 1 that are similar, i.e., for each stimuli, the HMMs are similar for all subjects in the group. Associated with each group are representative HMMs (i.e., cluster "centers"), one for each stimulus. Let $\mathcal{R}_j^{(s)}$ be the representative HMM for group j and stimulus s. Table 2 shows an example of co-clustering, where similar HMMs are grouped together to form representative HMMs.

	Stimulus 1	Stimulus 2
Subject 1	$\mathcal{B}_1^{(1)}$	$\mathcal{B}_1^{(2)}$
Subject 2	$\mathcal{B}_2^{(1)}$	$\mathcal{B}_2^{(2)}$
Subject 3	$\mathcal{B}_3^{(1)}$	$\mathcal{B}_3^{(2)}$
Subject 4	$\mathcal{B}_4^{(1)}$	$\mathcal{B}_4^{(2)}$

Table 1: Table of individual HMMs for subjects and stimuli.

	Stimulus 1	Stimulus 2			Stimulus 1	Stimulus 2
Subject 1	$\mathcal{B}_1^{(1)}$	$\mathcal{B}_1^{(2)}$	$\Rightarrow$	Group 1	$\mathcal{R}_1^{(1)}$	$\mathcal{R}_1^{(2)}$
Subject 2	$\mathcal{B}_2^{(1)}$	$\mathcal{B}_2^{(2)}$				
Subject 3	$\mathcal{B}_3^{(1)}$	$\mathcal{B}_3^{(2)}$	$\Rightarrow$	Group 2	$\mathcal{R}_2^{(1)}$	$\mathcal{R}_2^{(2)}$
Subject 4	$\mathcal{B}_4^{(1)}$	$\mathcal{B}_4^{(2)}$				

Table 2: Co-clustering will group similar rows (i.e., group similar HMMs across stimuli), and form representative HMMs for each group.

A.2 Derivation

We will use the same notation as the VHEM paper (Coviello et al., 2014), but we will use $\mathcal{B}$ and $\mathcal{R}$ to represent the base and reduced HMM mixtures (H3Ms), and superscript $^{(s)}$ to represent the stimuli index s. The set of base H3Ms $\mathcal{B} = \{\mathcal{B}^{(1)}, \cdots, \mathcal{B}^{(S)}\}$ contains the base H3M for each stimulus s, $\mathcal{B}^{(s)}$, and likewise for the set of reduced H3Ms $\mathcal{R} = \{\mathcal{R}^{(1)}, \cdots, \mathcal{R}^{(S)}\}$, where S is the number of simuli. For stimulus s, the base H3M $\mathcal{B}^{(s)}$ is composed from the individuals' HMMs $\{\mathcal{B}_i^{(s)}\}_{i=1}^{K^{(b)}}$, while the reduced H3M $\mathcal{B}^{(s)}$ comprises the representative HMMs $\{\mathcal{R}_j^{(s)}\}_{j=1}^{K^{(r)}}$, where $K^{(b)}$ is the number of individuals and $K^{(r)}$ is the number of groups.

A.2.1 Formulation

For the s-th stimuli, the likelihood functions for observed sequence $\mathbf{y}^{(s)}$ under the base (individuals') H3M $\mathcal{B}^{(s)}$ and the reduced (groups') H3M $\mathcal{R}^{(s)}$ are

$$p(\mathbf{y}^{(s)}|\mathcal{B}^{(s)}) = \sum_{i=1}^{K^{(b)}} \omega_i^{(b)} p(\mathbf{y}^{(s)}|\mathcal{B}_i^{(s)}), \tag{1}$$

$$p(\mathbf{y}^{(s)}|\mathcal{R}^{(s)}) = \sum_{j=1}^{K^{(r)}} \omega_j^{(r)} p(\mathbf{y}^{(s)}|\mathcal{R}_j^{(s)}). \tag{2}$$

Note that the cluster priors $\omega_i^{(b)}$ and $\omega_j^{(r)}$ are the same for each stimulus s, since we have the same number of subjects for each stimulus, and the same group sizes for each stimulus.

The VHEM algorithm first draws a set of virtual samples from each base mixture component (Coviello et al., 2014). For stimulus s, let $Y_i^{(s)}$ be a set of $N_i = N\omega_i^{(b)}$ virtual samples drawn from the ith base component, $Y_i^{(s)} \sim_{iid} p(\mathbf{y}^{(s)}|\mathcal{B}_i^{(s)})$. For stimulus s, the reduced HMMs are obtained by maximizing the expected log-likelihood using the virtual samples on stimulus s,

$$\mathcal{J}(\mathcal{R}^{(s)}) = \sum_{i=1}^{K^{(b)}} \mathbb{E}_{\mathcal{B}_i^{(s)}} \left[\log p(Y_i^{(s)}|\mathcal{R}^{(s)}) \right]. \tag{3}$$

For co-clustering, we aggregate the expected log-likelihood of virtual samples from all stimuli $\{Y_i^{(s)}\}$. Since the virtual samples are independent, we have

$$\mathcal{J}(\mathcal{R}) = \sum_{i=1}^{K^{(b)}} \sum_{s=1}^{S} \mathbb{E}_{\mathcal{B}_i^{(s)}} \left[\log p(Y_i^{(s)}|\mathcal{R}^{(s)}) \right]. \tag{4}$$

Finally the reduced model $\mathcal{R}$ is estimated by maximizing the expected log-likelihood in (4),

$$\mathcal{R}^* = \operatorname*{argmax}_{\mathcal{R}} \mathcal{J}(\mathcal{R}). \tag{5}$$

A.2.2 Variational Lower Bound

As with the original formulation, (4) is analytically intractable to evaluate. Hence, we construct a variational lower bound by deriving a lower bound for each term in the sum,

$$\mathcal{J}(\mathcal{R}) = \sum_{i=1}^{K^{(b)}} \sum_{s=1}^{S} \mathbb{E}_{\mathcal{B}_i^{(s)}} \left[\log p(Y_i^{(s)}|\mathcal{R}^{(s)}) \right] \geq \sum_{i=1}^{K^{(b)}} \sum_{s=1}^{S} \mathcal{L}_{H3M}^{(s),i} \tag{6}$$

where $\mathcal{L}_{H3M}^{(s),i}$ is a variational lower bound for each term in the summation,

$$\mathcal{L}_{H3M}^{(s),i} = \max_{z_{ij}} \sum_j z_{ij} \left\{ \log \frac{\omega_j^{(r)}}{z_{ij}} + N_i \mathcal{L}_{HMM}^{(s),i,j} \right\}. \tag{7}$$

The term $\mathcal{L}_{HMM}^{(s),i,j}$ is the lower-bound of the expected log-likelihood between $\mathcal{B}_i^{(s)}$ and $\mathcal{R}_j^{(s)}$, which is analogous to the lower-bound $\mathcal{L}_{HMM}^{i,j}$ for each stimuli s in VHEM (Coviello et al., 2014). The lower-bound $\mathcal{L}_{HMM}^{(s),i,j}$ can be computed using a variational algorithm by Coviello

et al. (2014). The lower-bound of $\mathcal{J}(\mathcal{R})$ is then

$$\mathcal{J}(\mathcal{R}) \geq \sum_{i=1}^{K^{(b)}} \sum_{s=1}^{S} \mathcal{L}_{H3M}^{(s),i} \tag{8}$$

$$= \sum_{i=1}^{K^{(b)}} \sum_{s=1}^{S} \max_{z_{ij}} \sum_{j} z_{ij} \left\{ \log \frac{\omega_j^{(r)}}{z_{ij}} + N_i \mathcal{L}_{HMM}^{(s),i,j} \right\} \tag{9}$$

$$= \sum_{i=1}^{K^{(b)}} \max_{z_{ij}} \sum_{s=1}^{S} \sum_{j} z_{ij} \left\{ \log \frac{\omega_j^{(r)}}{z_{ij}} + N_i \mathcal{L}_{HMM}^{(s),i,j} \right\} \tag{10}$$

$$= \sum_{i=1}^{K^{(b)}} \max_{z_{ij}} \sum_{j} z_{ij} \sum_{s=1}^{S} \left\{ \log \frac{\omega_j^{(r)}}{z_{ij}} + N_i \mathcal{L}_{HMM}^{(s),i,j} \right\} \tag{11}$$

$$= \sum_{i=1}^{K^{(b)}} \max_{z_{ij}} \sum_{j} z_{ij} \left\{ S \log \frac{\omega_j^{(r)}}{z_{ij}} + N_i \sum_{s=1}^{S} \mathcal{L}_{HMM}^{(s),i,j} \right\} \tag{12}$$

$$= S \sum_{i=1}^{K^{(b)}} \max_{z_{ij}} \sum_{j} z_{ij} \left\{ \log \frac{\omega_j^{(r)}}{z_{ij}} + N_i \frac{1}{S} \sum_{s=1}^{S} \mathcal{L}_{HMM}^{(s),i,j} \right\}, \tag{13}$$

where in (10) the "max" operator can be swapped with the summation over s because z_{ij} is the same for each stimulus s.

Defining the term

$$L_{i,j} = \log \omega_j^{(r)} + N_i \frac{1}{S} \sum_{s=1}^{S} \mathcal{L}_{HMM}^{(s),i,j}, \tag{14}$$

the maximum with respect to the assignment variables z_{ij} is obtained using results from Appendix D.2 of Coviello et al. (2014),

$$\hat{z}_{ij} = \frac{\exp L_{i,j}}{\sum_{j'} \exp L_{i,j'}} = \frac{\omega_j^{(r)} \exp\left(N_i \frac{1}{S} \sum_{s=1}^{S} \mathcal{L}_{HMM}^{(s),i,j} \right)}{\sum_{j'} \omega_{j'}^{(r)} \exp\left(N_i \frac{1}{S} \sum_{s=1}^{S} \mathcal{L}_{HMM}^{(s),i,j'} \right)}. \tag{15}$$

The main difference between the assignment variable in (15) and the corresponding Eq. 19 in the VHEM paper (Coviello et al., 2014) is that (15) averages the expected log-likelihoods between HMMs $\mathcal{L}_{HMM}^{(s),i,j}$ over all the stimuli s.

After calculating the assignment variables $\hat{z}_{ij}$, the remaining E-step is equivalent to using the original E-step of VHEM on each stimulus separately. That is, the summary statistics $\Theta_{ij}^{(s)}$ are computed separately for each stimulus s. The M-step then updates the models using the shared assignment variables $\hat{z}_{ij}$ for each stimulus s. Finally, convergence

is tested using the lower bound of $\mathcal{J}(\mathcal{R})$, by substituting the optimal $\hat{z}_{ij}$ into (13),

$$
\begin{aligned}
\mathcal{J}(\mathcal{R}) &\geq S \sum_i \sum_j \hat{z}_{ij}(L_{i,j} - \log \hat{z}_{ij}) && (16)\\
&= S \sum_i \sum_j \hat{z}_{ij}(L_{i,j} - [L_{ij} - \log \sum_{j'} \exp(L_{ij'})]) && (17)\\
&= S \sum_i \sum_j \hat{z}_{ij} \log \sum_{j'} \exp(L_{ij'}) && (18)\\
&= S \sum_i (\sum_j \hat{z}_{ij}) \log \sum_{j'} \exp(L_{ij'}) && (19)\\
&= S \sum_i \log \sum_{j'} \exp(L_{ij'}), && (20)
\end{aligned}
$$

where the last line follows from the property that assignment variables sum to 1 (over j).

A.2.3 VHEM Algorithm with co-clustering

The VHEM algorithm with co-clustering is summarized in Algorithm 1. Except for the calculation of the assignment variables $\hat{z}_{ij}$, the algorithm is based on steps from the original VHEM algorithm by Coviello et al. (2014). The notation (see Coviello et al., 2014, Alg. 1, L7-11) refers to lines 7-11 of the original VHEM algorithm (Algorithm 1) by Coviello et al. (2014).

Algorithm 1 HEM algorithm for co-clustering

Input: Set of subject HMMs for various stimuli $\{\{\mathcal{B}_i^{(s)}\}_{s=1}^S\}_{i=1}^{K^{(b)}}$. S is the number of stimuli. $K^{(b)}$ is the number of base HMMs. $K^{(r)}$ is the number of reduced HMMs.
Initialize group HMMs for each stimuli: $\mathcal{R}_j^{(s)}$ for each j and s.
repeat
  {E-step}
  for $s = \{1, \ldots, S\}$ and $i = \{1, \ldots, K^{(b)}\}$ and $j = \{1, \ldots, K^{(r)}\}$ **do**
    Calculate expected log-likelihood $\mathcal{L}_{HMM}^{(s),i,j}$ (see Coviello et al., 2014, Alg. 1, L7-11).
    Calculate summary statistics $\Theta_{i,j}^{(s)}$ (see Coviello et al., 2014, Alg. 1, L13).
  end for
  Calculate assignment variables $\hat{z}_{ij}$ using (14) and (15).
  {M-step}
  for $s = \{1, \ldots, S\}$ and $j = \{1, \ldots, K^{(r)}\}$ **do**
    Compute group model $\mathcal{R}_j^{(s)}$ using statistics $\{\Theta_{i,j}^{(s)}\}_{i=1}^{K^{(b)}}$ and assignment variables $\{\hat{z}_{ij}\}_{i=1}^{K^{(b)}}$ (see Coviello et al., 2014, Alg. 1, L15).
  end for
until convergence.
Output: Set of group HMMs for each stimuli $\{\{\mathcal{R}_j^{(s)}\}_{s=1}^S\}_{j=1}^{K^{(r)}}$. Group assignment probabilities $\hat{z}_{ij}$.

B. Derivation of VBHEM with Co-clustering

In this Appendix we provide the formal derivation of VBHEM with co-clustering. The original variational bayesian hierarchical EM (VBHEM) algorithm is a bayesian version of variational hierarchical EM (VHEM) algorithm (Coviello et al., 2014), which clusters a set of individuals' HMMs into groups and forms a representative HMM for each group. Here we modify VBHEM to perform co-clustering over several sets of HMMs, where each set corresponds to individuals' HMMs for one stimulus. In particular, VBHEM with co-clustering is equivalent to running VBHEM separately on each set of HMMs (i.e., for each stimulus), but it computes consistent cluster assignments of individuals to groups across all stimuli (i.e., all runs of VBHEM). The output result of co-clustering is a set of representative HMMs (one for each stimulus) for each group. The main difference between VBHEM and VHEM is that VBHEM assumes prior distributions on the HMM parameters of the group models. The number of groups can be estimated by maximizing the marginal likelihood of the data.

B.1 Problem Formulation

The co-clustering problem formulation and notation is the same as the VHEM derivation above. In particular, we use $\mathcal{B}$ and $\mathcal{R}$ to represent the base and reduced HMM mixtures (H3Ms), and superscript (s) to represent the stimuli index s. The set of base H3Ms $\mathcal{B} = \{\mathcal{B}^{(1)}, \cdots, \mathcal{B}^{(S)}\}$ contains the base H3M for each stimulus s, $\mathcal{B}^{(s)}$, and likewise for the set of reduced H3Ms $\mathcal{R} = \{\mathcal{R}^{(1)}, \cdots, \mathcal{R}^{(S)}\}$, where S is the number of stimuli. For stimulus s, the base H3M $\mathcal{B}^{(s)}$ is composed from the individuals' HMMs $\{\mathcal{B}_i^{(s)}\}_{i=1}^{K^{(s,b)}}$, while the reduced H3M $\mathcal{R}^{(s)}$ comprises the representative HMMs $\{\mathcal{R}_j^{(s)}\}_{j=1}^{K^{(r)}}$, where $K^{(s,b)}$ is the number of individuals viewing stimulus s and $K^{(r)}$ is the number of groups.

B.2 Evidence Lower Bound

While VHEM considers the parameters of reduced model $\mathcal{R}_j^{(s)}$ as unknown terms and computes its point-estimate, VBHEM considers the parameters of reduced mode $\mathcal{R}_j^{(s)}$ as hidden variables, and gives priors for each parameters and computes its posteriors. Given the observations $\boldsymbol{Y}$ and hidden variables $\boldsymbol{H}$. The VB framework for H3M with K components and S states is formulated as follows. Starting from the *marginal log-likelihood* (i.e., model evidence) $\log p(\boldsymbol{Y})$,

$$\log p(\boldsymbol{Y}) = \mathcal{L}(q) + \mathrm{KL}(q||p)$$

where we have defined

$$\mathcal{L}(q) = \sum_{K,S} q(K,S)\Big[\mathcal{L}_{(K,S)}(q) + \log\frac{p(K,S)}{q(K,S)}\Big], \tag{21}$$

$$\mathcal{L}_{(K,S)}(q) = \int q(\boldsymbol{H}|K,S)\log\frac{p(\boldsymbol{Y},\boldsymbol{H}|K,S)}{q(\boldsymbol{H}|K,S)}d\boldsymbol{H}, \tag{22}$$

$$\mathrm{KL}(q||p) = \sum_{K,S}\int q(\boldsymbol{H},K,S)\log\frac{q(\boldsymbol{H},K,S)}{p(\boldsymbol{H},K,S|\boldsymbol{Y})}d\boldsymbol{H},$$

and $\mathrm{KL}(q||p) = \int q(z)\log\frac{q(z)}{p(z)}dz$ is Kullback-Leibler divergence (KLD) between distributions q and p, and satisfies $\mathrm{KL}(q||p)) \geq 0$. Thus, we have $\log p(\boldsymbol{Y}) \geq \mathcal{L}(q)$, which holds for any distribution $q(\boldsymbol{H},K,S)$, and equality occurs when $q(\boldsymbol{H},K,S) = p(\boldsymbol{H},K,S|\boldsymbol{Y})$ (i.e. $\mathrm{KL}(q||p)) = 0$). Therefore, $\mathcal{L}(q)$ is a lower bound on $\log p(\boldsymbol{Y})$, and optimizing $\mathcal{L}(q)$ w.r.t $q(\boldsymbol{H},K,S)$ will obtain an approximation of the true posterior distribution $p(\boldsymbol{H},K,S|\boldsymbol{Y})$.

However, if we maximize $\mathcal{L}(q)$ w.r.t. $q(\boldsymbol{H}|K,S)$, the results for different pairs of (K,S) are coupled since they are conditioned on K and S. We proceed instead by first optimizing each of the $q(\boldsymbol{H}|K,S)$ individually by optimization of $\mathcal{L}_{(K,S)}(q)$. Assuming that $q(\boldsymbol{H}|K,S) = \prod_l^L q_l(H_l|K,S)$ and $\{H_l\}_{l\in[L]}$ is a partition of $\boldsymbol{H}$. Then, the optimal solution $q_l^*(H_l|K,S)$ is given by (Bishop, 2006):

$$\log q_l^*(H_l|K,S) = \mathbb{E}_{l'\neq l}[\log p(\boldsymbol{Y},\boldsymbol{H}|K,S)] + \text{const}, \tag{23}$$

$$\mathbb{E}_{l'\neq l}[\log p(\boldsymbol{Y},\boldsymbol{H}|K,S] = \int(\log p(\boldsymbol{Y},\boldsymbol{H}|K,S))\prod_{l'\neq l} q_{l'}(H_{l'}|K,S)dH_l'.$$

Model Selection. For a set of candidate models, i.e. different pairs of (K,S), we can rewrite (21) as

$$\mathcal{L}(q) = \sum_{K,S} q(K,S)\Big[\log\big(p(K,S)\exp\{\mathcal{L}_{(K,S)}(q)\}\big) - \log q(K,S)\Big]. \tag{24}$$

Recognizing (24) as the negative KLD between $q(K,S)$ and the distribution (not necessarily normalized) $p(K,S)\exp\{\mathcal{L}_{(K,S)}(q)\}$. The lower bound $\mathcal{L}(q)$ will be maximized when the KLD is minimized, which will be the case when

$$q^*(K,S) \propto p(K,S)\exp\left\{\mathcal{L}_{(K,S)}(q)\right\}$$

Thus, the optimal model structure can be found as $(K^*,S^*) = \underset{K,S}{\mathrm{argmax}}\, q^*(K,S)$.

B.3 Formulation

Formally, let $\mathcal{B}^{(s)} = \{\omega_i^{(s,b)}, \mathcal{B}_i^{(s)}\}_{i=1}^{K^{(s,b)}}$ represents a "base" hidden Markov model mixtures (H3M) for the s-th stimulus with $K^{(s,b)}$ components (HMMs) and $S^{(b)}$ states for each component. The reduced model for s-th stimulus is denoted by $\mathcal{R}^{(s)} = \{\omega_j^{(r)}, \mathcal{R}_j^{(s)}\}_{j=1}^{K^{(r)}}$ with $S^{(r)}$ states, where $K^{(s,b)} > K^{(r)}$ and $S^{(b)} \geq S^{(r)}$. Note that in the co-clustering setting,

the different stimuli share the same mixture weight $\omega_j^{(r)}$. In the Bayesian framework, we assume priors on all unknown parameters in $\mathcal{R}^{(s)}$,

$$\begin{aligned}
&p(\boldsymbol{\Omega}^{(r)}) = \text{Dir}(\boldsymbol{\Omega}^{(r)}|\boldsymbol{\alpha}_0)\\
&p(\boldsymbol{\pi}_j^{(s,r)}) = \text{Dir}(\boldsymbol{\pi}_j^{(s,r)}|\boldsymbol{\eta}_0),\\
&p(\boldsymbol{A}_j^{(s,r)}) = \prod_\rho p(\boldsymbol{a}_{j,\rho}^{(s,r)}) = \prod_\rho \text{Dir}(\boldsymbol{a}_{j,\rho}^{(s,r)}|\boldsymbol{\epsilon}_0),\\
&p(\boldsymbol{\mu}_j^{(s,r)}, \boldsymbol{\Lambda}_j^{(s,r)}) = \prod_\rho p(\boldsymbol{\mu}_{j,\rho}^{(s,r)}, \boldsymbol{\Lambda}_{j,\rho}^{(s,r)})\\
&p(\boldsymbol{\mu}_{j,\rho}^{(s,r)}, \boldsymbol{\Lambda}_{j,\rho}^{(s,r)}) = \mathcal{N}(\boldsymbol{\mu}_{j,\rho}^{(s,r)}|\boldsymbol{m}_0, (\gamma_0\boldsymbol{\Lambda}_{j,\rho}^{(s,r)})^{-1}) \cdot \mathcal{W}(\boldsymbol{\Lambda}_{j,\rho}^{(s,r)}|\boldsymbol{W}_0, \nu_0),
\end{aligned}$$

where $\text{Dir}(\cdot|\boldsymbol{\alpha})$ is a Dirichlet distribution with concentration vector $\boldsymbol{\alpha}$, $\mathcal{N}(\cdot|\boldsymbol{\mu}, \boldsymbol{\Sigma})$ is a Gaussian distribution with mean $\boldsymbol{\mu}$ and covariance $\boldsymbol{\Sigma}$, and $\mathcal{W}(\cdot|\boldsymbol{W}, \nu)$ is a Wishart distribution with scale matrix $\boldsymbol{W}$ and degrees-of-freedom ν.

We also assume prior distributions over the number of component $K^{(r)}$ and states $S^{(r)}$ in the reduced model as uniform distributions on $K^{(r)}$ and $S^{(r)}$,

$$\begin{aligned}
p(K^{(r)}) &= \frac{1}{K_{max}^{(r)} - K_{min}^{(r)} + 1}, \qquad K_{min}^{(r)} \leq K^{(r)} \leq K_{max}^{(r)}.\\
p(S^{(r)}) &= \frac{1}{S_{max}^{(r)} - S_{min}^{(r)} + 1}, \qquad S_{min}^{(r)} \leq S^{(r)} \leq S_{max}^{(r)},
\end{aligned}$$

where $(K_{min}^{(r)}, K_{max}^{(r)})$ and $(S_{min}^{(r)}, S_{max}^{(r)})$ are the minimum and maximum possible values that $K^{(r)}$ and $S^{(r)}$ can take. Table 3 summarizes the notation used in the derivation, including the variable names, latent variables, model names, and hyperparameters. The algorithm is summarized is Algorithm 2.

In order to compute the posteriors of each parameters using (23), we first consider the fixed $K^{(r)}$ and $S^{(r)}$ and give the joint probability distribution as

$$\log p(\boldsymbol{Y}, \boldsymbol{Z}, \mathcal{R}, \boldsymbol{\Omega}^{(r)}) = \log p(\boldsymbol{Y}|\boldsymbol{Z}, \mathcal{R}) + \log p(\boldsymbol{Z}|\boldsymbol{\Omega}^{(r)}) + p(\mathcal{R}) + p(\boldsymbol{\Omega}^{(\boldsymbol{r})})$$

where the hidden variables $\boldsymbol{H} = \{\boldsymbol{Z}, \boldsymbol{\Omega}^{(r)}, \mathcal{R}\}$. For each term,

1. term $\log p(\boldsymbol{Y}|\boldsymbol{Z}, \mathcal{R})$:

$$\begin{aligned}
\log p(\boldsymbol{Y}|\boldsymbol{Z}, \mathcal{R}) &= \sum_i \sum_s \log p(\boldsymbol{Y}_i^{(s)}|\boldsymbol{Z}_i^{(s)}, \mathcal{R}^{(s)})\\
&= \sum_i \sum_s \sum_j z_{i,j}^{(s)} \log p(\boldsymbol{Y}_i^{(s)}|\mathcal{R}_j^{(s)})\\
&= \sum_i \sum_s \sum_j z_{i,j}^{(s)} N_i^{(s)} \mathbb{E}_{\boldsymbol{y}|\mathcal{B}_i^{(s)}} \log p(\boldsymbol{y}|\mathcal{R}_j^{(s)})\\
&= \sum_i \sum_s \sum_j z_{i,j}^{(s)} N_i^{(s)} \mathcal{L}_{HMM}^{(s),i,j}
\end{aligned}$$

Algorithm 2 VBHEM algorithm for co-clustering

Input: Set of subject HMMs for various stimuli $\{\{\mathcal{B}_i^{(s)}\}_{i=1}^{K^{(s,b)}}\}_{s=1}^{S}$, hyperparameters set $\mathcal{H}^{(r)}$, the number of cluster $K^{(r)} \in \{K_{min}^{(r)}, ..., K_{max}^{(r)}\}$ and the number of states $S^{(r)} \in \{S_{min}^{(r)}, ..., S_{max}^{(r)}\}$, the number of virtual samples N.
Output: reduced H3M $\mathcal{R} = \{\{\mathcal{R}_j^{(s)}\}_{j=1}^{K^{(r)}}\}_{s=1}^{S}$.

for each $(K^{(r)}, S^{(r)})$ **do**
  repeat
    {E-step}
    Compute responsibilities $\hat{z}_{i,j}$ using (26).
    {M-step}
    for $s = \{1, ..., S\}$ and $j = \{1, ..., K^{(r)}\}$ **do**
      Update the hyperparameters α_j, $\eta_{j,\rho}^{(s)}$, $\boldsymbol{\epsilon}_{j,\rho}^{(s)}$, $\boldsymbol{m}_{j,\rho}^{(s)}$, $\gamma_{j,\rho}^{(s)}$, $\boldsymbol{W}_{j,\rho}^{(s)}$, and $\nu_{j,\rho}^{(s)}$ for each state ρ by (27), (33) , (35) and (29 - 32), respectively.
    end for
  until convergence of $\mathcal{L}_{(K^{(r)},S^{(r)})}(q^*)$.
end for
Select the reduced H3M $\mathcal{R}$ with maximum $\mathcal{L}_{(K^{(r)},S^{(r)})}(q^*)$.
Compute the posterior expectation of each parameter to get reduced model $\mathcal{R}^{(s)}, s = 1, ..., S$.

where $N_i^{(s)} = N^{(s)}\omega_i^{(s,b)}$ is the number of virtual sample for each subject i under stimuli s. We defined that $N^{(s)} \equiv N$ and $\omega_i^{(s,b)} = 1/K^{(s,b)}$, where $K^{(s,b)}$ is the number of subjects viewing the stimulus s. Note that we also assume that $z_{i,j}^{(s)} = z_{i,j}$, i.e., the cluster assignments are the same among all stimuli, which is based on the co-clustering framework. Also, we have defined,

$$
\begin{aligned}
\mathcal{L}_{HMM}^{(s),i,j} &= \mathbb{E}_{\boldsymbol{y}|\mathcal{B}_i^{(s)}} \log p(\boldsymbol{y}|\mathcal{R}_j^{(s)}) \\
&\geq \sum_{\boldsymbol{\beta}} \boldsymbol{\pi}_{i,\boldsymbol{\beta}}^{(s,b)} \sum_{\boldsymbol{\rho}} \phi^{(s),i,j}(\boldsymbol{\rho}|\boldsymbol{\beta}) \cdot \Big[\log \frac{\pi_{j,\boldsymbol{\rho}}^{(s,r)}}{\phi^{(s),i,j}(\boldsymbol{\rho}|\boldsymbol{\beta})} + \sum_{t=1}^{\tau} \mathbb{E}_{\boldsymbol{y}|\boldsymbol{\Theta}_{i,\beta_t}^{(s,b)}} \log p(\boldsymbol{y}|\boldsymbol{\Theta}_{j,\rho_t}^{(s,r)}) \Big],
\end{aligned}
$$

where $\phi^{(s),i,j}(\boldsymbol{\rho}|\boldsymbol{\beta}) = \phi_1^{(s),i,j}(\rho_1|\beta_1) \sum_{t=2}^{\tau} \phi_t^{(s),i,j}(\rho_t|\rho_{t-1}, \beta_t)$, satisfying that $\sum_{\rho_1=1}^{S^{(r)}} \phi_1^{(s),i,j}(\rho_1|\beta_1) = 1$, $\sum_{\rho_t=1}^{S^{(r)}} \phi_t^{(s),i,j}(\rho_t|\rho_{t-1}, \beta_t) = 1$, and all the factors are non-negative.

2. term $\log p(\boldsymbol{Z}|\boldsymbol{\Omega}^{(r)})$:

$$
\begin{aligned}
\log p(\boldsymbol{Z}|\boldsymbol{\Omega}^{(r)}) &= \sum_i \sum_s \sum_j z_{i,j} N_i^{(s)} \log \omega_j^{(r)} \\
&= \sum_i \sum_j z_{i,j} \big[\sum_s N_i^{(s)} \big] \log \omega_j^{(r)}
\end{aligned}
$$

Table 3: Notation used in the derivation of the VBHEM Co-clustering Algorithm. s refers to the stimulus number.

variables	*base model (b)*	*reduced model (r)*
number of HMM components	$K^{(s,b)}$	$K^{(r)}$
index for HMM components	i	j
number of HMM states	$S^{(b)}$	$S^{(r)}$
index for HMM states	β	ρ
HMM state sequence	$\boldsymbol{\beta} = \{\beta_t\}_{t=1}^{\tau}$	$\boldsymbol{\rho} = \{\rho_t\}_{t=1}^{\tau}$
models		
H3M	$\mathcal{B}^{(s)} = \{\boldsymbol{\Omega}^{(s,b)}, \{\mathcal{B}_i^{(s)}\}_{i=1}^{K^{(s,b)}}\}$	$\mathcal{R}^{(s)} = \{\boldsymbol{\Omega}^{(r)}, \{\mathcal{R}_j^{(s)}\}_{j=1}^{K^{(r)}}\}$
HMM component (of H3M)	$\mathcal{B}_i^{(s)} = \{\boldsymbol{\pi}_i^{(s,b)}, \boldsymbol{A}_i^{(s,b)}, \{\boldsymbol{\Theta}_{i,\beta}^{(s,b)}\}_{\beta=1}^{S^{(b)}}\}$	$\mathcal{R}_j^{(s)} = \{\boldsymbol{\pi}_j^{(s,r)}, \boldsymbol{A}_j^{(s,r)}, \{\boldsymbol{\Theta}_{j,\rho}^{(s,r)}\}_{\rho=1}^{S^{(r)}}\}$
Gaussian emission	$\boldsymbol{\Theta}_{i,\beta}^{(s,b)} = \{\boldsymbol{\mu}_{i,\beta}^{(s,b)}, \boldsymbol{\Lambda}_{i,\beta}^{(s,b)}\}$	$\boldsymbol{\Theta}_{j,\rho}^{(s,r)} = \{\boldsymbol{\mu}_{j,\rho}^{(s,r)}, \boldsymbol{\Lambda}_{j,\rho}^{(s,r)}\}$
latent variables		
assignment variable	$\boldsymbol{Z}'$	$\boldsymbol{Z}$
hidden state sequence	$\boldsymbol{X}'$	$\boldsymbol{X}$
H3M mixture weights	$\boldsymbol{\Omega}^{(s,b)} = \{\omega_i^{(s,b)}\}$	$\boldsymbol{\Omega}^{(r)} = \{\omega_j^{(r)}\}$
HMM initial probability	$\boldsymbol{\pi}_i^{(s,b)} = \{\pi_{i,\beta}^{(s,b)}\}$	$\boldsymbol{\pi}_j^{(s,r)} = \{\pi_{j,\rho}^{(s,r)}\}$
HMM state transition matrix	$\boldsymbol{A}_i^{(s,b)} = [a_{i,\beta,\beta'}^{(s,b)}]$	$\boldsymbol{A}_j^{(s,r)} = [a_{j,\rho,\rho'}^{(s,r)}]$
Gaussian emission	$\{\boldsymbol{\mu}_{i,\beta}^{(s,b)}, (\boldsymbol{\Lambda}_{i,\beta}^{(s,b)})^{-1}\}$	$\{\boldsymbol{\mu}_{j,\rho}^{(s,r)}, (\boldsymbol{\Lambda}_{j,\rho}^{(s,r)})^{-1}\}$
hyperparameters		*reduced model (r)*
hyperparameters		$\mathcal{H}^{(r)} = \{\alpha_0, \eta_0, \epsilon_0, \gamma_0, \boldsymbol{m}_0, \boldsymbol{W}_0, \nu_0\}$

3. term $\log p(\boldsymbol{\Omega}^{(r)})$:

$$\begin{aligned}\log p(\boldsymbol{\Omega}^{(r)}) &= \log \mathrm{Dir}(\boldsymbol{\Omega}^{(r)}|\alpha_0)\\ &= \log C(\alpha_0) + (\alpha_0 - 1)\sum_j \log \omega_j^{(r)}\end{aligned}$$

4. term $\log p(\mathcal{R})$:

$$\log p(\mathcal{R}) = \sum_j \sum_s \left[\log p(\boldsymbol{\pi}_j^{(s,r)}) + \log p(\boldsymbol{A}_j^{(s,r)}) + \log p(\boldsymbol{\mu}_j^{(s,r)}, \boldsymbol{\Lambda}_j^{(s,r)})\right]$$

B.4 Optimal Variational Distribution

The hidden variables in our model are the assignment variables $\boldsymbol{Z}$, the group HMMs $\mathcal{R}$, and the group HMMs component weights $\boldsymbol{\Omega}^{(r)}$. The group HMMs $\mathcal{R}$ consist of HMM parameters $\{\boldsymbol{\pi}_j^{(s,r)}, \boldsymbol{A}_j^{(s,r)}, \boldsymbol{\mu}_j^{(s,r)}, \boldsymbol{\Lambda}_j^{(s,r)}\}$ for each stimuli s and group j. We assume that the

approximate variational posterior factorizes according to

$$q(\boldsymbol{Z}, \boldsymbol{\Omega^{(r)}}, \mathcal{R}) = q(\boldsymbol{Z})q(\boldsymbol{\Omega^{(r)}}) \prod_{j,s} q(\boldsymbol{\pi}_j^{(s,r)})q(\boldsymbol{A}_j^{(s,r)})q(\boldsymbol{\mu}_j^{(s,r)}, \boldsymbol{\Lambda}_j^{(s,r)}). \tag{25}$$

The optimal variational distribution q^* are found via (23). For each hidden variable, we obtain the posterior:

1. $\log q^*(\boldsymbol{Z})$

$$\begin{aligned}\log q^*(\boldsymbol{Z}) &= \mathbb{E}_{\{\mathcal{R}, \boldsymbol{\Omega}^{(r)}\}}[\log p(\boldsymbol{Y}, \boldsymbol{Z}, \mathcal{R}, \boldsymbol{\Omega}^{(r)})] + const \\ &\propto \mathbb{E}_{\{\mathcal{R}\}} \log p(\boldsymbol{Y}|\boldsymbol{Z}, \mathcal{R}) + \mathbb{E}_{\{\boldsymbol{\Omega}^{(r)}\}} \log p(\boldsymbol{Z}|\boldsymbol{\Omega}^{(r)}) \\ &= \sum_i \sum_s \sum_j z_{i,j} N_i^{(s)} \mathbb{E}_{\{\mathcal{R}\}} \mathcal{L}_{HMM}^{(s),i,j} + \sum_s \sum_i \sum_j z_{i,j} N_i^{(s)} \mathbb{E}_{\{\boldsymbol{\Omega}^{(r)}\}} \log \omega_j^{(r)} \\ &= \sum_i \sum_j z_{i,j} \Big[\big[\sum_s N_i^{(s)} \big] \log \tilde{\omega}_j^{(r)} + \sum_s N_i^{(s)} \tilde{\mathcal{L}}_{HMM}^{(s),i,j} \Big] \\ &= \sum_i \sum_j z_{i,j} \log r_{i,j}.\end{aligned}$$

Normalizing $z_{i,j}$,

$$\hat{z}_{i,j} = \frac{r_{i,j}}{\sum_l r_{i,l}} = \frac{\exp\{ \big[\sum_s N_i^{(s)} \big] \log \tilde{\omega}_j^{(r)} + \sum_s N_i^{(s)} \tilde{\mathcal{L}}_{HMM}^{(s),i,j} \}}{\sum_l \exp\{ \big[\sum_s N_i^{(s)} \big] \log \tilde{\omega}_l^{(r)} + \sum_s N_i^{(s)} \tilde{\mathcal{L}}_{HMM}^{(s),i,l} \}}, \tag{26}$$

where the $\hat{z}_{i,j}$ is the probability that i-th subject is assigned to j-th Group. Also, we have defined,

$$\begin{aligned}\tilde{\mathcal{L}}_{HMM}^{(s),i,j} &= \mathbb{E}_{\mathcal{R}_j^{(s)}}[\mathcal{L}_{HMM}^{(s),i,j}] \\ &= \max_{\phi^{(s),i,j}} \sum_{\boldsymbol{\beta}} \boldsymbol{\pi}_{i,\boldsymbol{\beta}}^{(s,b)} \sum_{\boldsymbol{\rho}} \phi^{(s),i,j}(\boldsymbol{\rho}|\boldsymbol{\beta}) \Big[\log \frac{\tilde{\pi}_{j,\boldsymbol{\rho}}^{(s,r)}}{\phi^{(s),i,j}(\boldsymbol{\rho}|\boldsymbol{\beta})} + \sum_{t=1}^{\tau} \mathbb{E}_{\boldsymbol{\Theta}_{j,\rho_t}^{(s,r)}} \mathbb{E}_{\boldsymbol{y}|\boldsymbol{\Theta}_{i,\beta_t}^{(s,b)}} \log p(\boldsymbol{y}|\boldsymbol{\Theta}_{j,\rho_t}^{(s,r)}) \Big],\end{aligned}$$

where,

$$\begin{aligned}\log \tilde{\pi}_{j,\boldsymbol{\rho}}^{(s,r)} &= \log \tilde{\pi}_{j,\rho_1}^{(s,r)} + \sum_{t=2}^{\tau} \log \tilde{a}_{j,\rho_{t-1},\rho_t}^{(s,r)} \\ \log \tilde{\pi}_{j,\rho_1}^{(s,r)} &\triangleq \mathbb{E}_{\boldsymbol{\pi}_j^{(s,r)}}[\log \pi_{j,\rho_1}^{(s,r)}], \qquad \log \tilde{a}_{j,\rho_{t-1},\rho_t}^{(s)} \triangleq \mathbb{E}_{\boldsymbol{a}_j^{(s)}}[\log a_{j,\rho_{t-1},\rho_t}^{(s)}].\end{aligned}$$

2. $\log q^*(\boldsymbol{\Omega}^{(r)})$

$$\begin{aligned}\log q(\boldsymbol{\Omega}^{(r)}) &\propto \mathbb{E}_{\boldsymbol{Z}} \log p(\boldsymbol{Z}|\boldsymbol{\Omega}^{(r)}) + \log p(\boldsymbol{\Omega}^{(r)}) \\ &= \sum_i \sum_j \hat{z}_{i,j} \big[\sum_s N_i^{(s)} \big] \log \omega_j^{(r)} + (\alpha_0 - 1) \sum_j \log \omega_j^{(r)} \\ &= \sum_j \Big[\sum_i \hat{z}_{i,j} \big[\sum_s N_i^{(s)} \big] + \alpha_0 - 1 \Big] \log \omega_j^{(r)}.\end{aligned}$$

Thus,

$$\alpha_j = \alpha_0 + N_j \tag{27}$$

$$N_j = \sum_i \hat{z}_{i,j}\big[\sum_s N_i^{(s)}\big] \tag{28}$$

and N_j is the number of samples that have been assigned to $\mathcal{R}_j^{(s)}$.

3. $\log q^*(\boldsymbol{\mu}^{(r)}, \boldsymbol{\Lambda}^{(r)})$

$$\begin{aligned}
\log q(\boldsymbol{\mu}^{(r)}, \boldsymbol{\Lambda}^{(r)}) &\propto \mathbb{E}_{\boldsymbol{Z}} \log p(\boldsymbol{Y}|\boldsymbol{Z}, \mathcal{R}) + \log p(\boldsymbol{\mu}^{(r)}, \boldsymbol{\Lambda}^{(r)}) \\
&= \sum_s \sum_i \sum_j \hat{z}_{i,j} N_i^{(s)} \mathcal{L}_{HMM}^{(s),i,j} + \sum_s \sum_j \log p(\boldsymbol{\mu}_j^{(s,r)}, \boldsymbol{\Lambda}_j^{(s,r)}) \\
&= \sum_s \sum_j \Big[\sum_i \hat{z}_{i,j} N_i^{(s)} \mathcal{L}_{HMM}^{(s),i,j} + \log p(\boldsymbol{\mu}_j^{(s,r)}, \boldsymbol{\Lambda}_j^{(s,r)})\Big] \\
&= \sum_s \sum_j \log q^*(\boldsymbol{\mu}_j^{(s,r)}, \boldsymbol{\Lambda}_j^{(s,r)}).
\end{aligned}$$

Removing the terms in $\mathcal{L}_{HMM}^{(s),i,j}$ that irrelevant to the mean and covariance,

$$\begin{aligned}
&\log q^*(\boldsymbol{\mu}_j^{(s,r)}, \boldsymbol{\Lambda}_j^{(s,r)}) \\
&= \sum_i \hat{z}_{i,j} N_i^{(s)} \sum_{\boldsymbol{\beta}} \pi_{i,\boldsymbol{\beta}}^{(s,b)} \sum_{\boldsymbol{\rho}} \phi^{(s),i,j}(\boldsymbol{\rho}|\boldsymbol{\beta}) \sum_t \mathbb{E}_{y|\mathcal{B}_{i,\beta_t}^{(s)}} \log p(y|\mathcal{R}_{j,\rho_t}^{(s)}) + \log p(\boldsymbol{\mu}_j^{(s,r)}, \boldsymbol{\Lambda}_j^{(s,r)}) \\
&= \sum_i \hat{z}_{i,j} N_i^{(s)} \sum_\beta \sum_\rho \hat{\nu}^{(s),i,j}(\rho, \beta) \mathbb{E}_{y|\mathcal{B}_{i,\beta}^{(s)}} \log p(y|\mathcal{R}_{j,\rho}^{(s)}) + \sum_\rho \log p(\boldsymbol{\mu}_{j,\rho}^{(s,r)}, \boldsymbol{\Lambda}_{j,\rho}^{(s,r)}) \\
&= \sum_\rho \Big[\sum_i \hat{z}_{i,j} N_i^{(s)} \sum_\beta \hat{\nu}^{(s),i,j}(\rho, \beta) \mathbb{E}_{y|\mathcal{B}_{i,\beta}^{(s)}} \log p(y|\mathcal{R}_{j,\rho}^{(s)}) + \log p(\boldsymbol{\mu}_{j,\rho}^{(s,r)}, \boldsymbol{\Lambda}_{j,\rho}^{(s,r)})\Big] \\
&= \sum_\rho \log q^*(\boldsymbol{\mu}_{j,\rho}^{(s,r)}, \boldsymbol{\Lambda}_{j,\rho}^{(s,r)}).
\end{aligned}$$

Then,

$$q^*(\boldsymbol{\mu}_{j,\rho}^{(s,r)}, \boldsymbol{\Lambda}_{j,\rho}^{(s,r)}) = \mathcal{N}(\boldsymbol{\mu}_{j,\rho}^{(s,r)}|\boldsymbol{m}_{j,\rho}^{(s,r)}, (\gamma_{j,\rho}^{(s,r)}\boldsymbol{\Lambda}_{j,\rho}^{(s,r)})^{-1})\mathcal{W}(\boldsymbol{\Lambda}_{j,\rho}^{(s,r)}|\boldsymbol{W}_{j,\rho}^{(s,r)}, \nu_{j,\rho}^{(s,r)}),$$

where

$$\gamma_{j,\rho}^{(s)} = \gamma_0 + N_{j,\rho}^{(s)}, \tag{29}$$

$$\boldsymbol{m}_{j,\rho}^{(s)} = \frac{1}{\gamma_{j,\rho}^{(s)}}(\gamma_0 \boldsymbol{m}_0 + N_{j,\rho}^{(s)}\bar{\boldsymbol{y}}_{j,\rho}^{(s)}), \tag{30}$$

$$(\boldsymbol{W}_{j,\rho}^{(s)})^{-1} = \boldsymbol{W}_0^{-1} + N_{j,\rho}^{(s)}\boldsymbol{S}_{j,\rho}^{(s)} + N_{j,\rho}^{(s)}\boldsymbol{C}_{j,\rho}^{(s)} + \frac{\gamma_0 N_{j,\rho}^{(s)}}{\gamma_0 + N_{j,\rho}^{(s)}}(\bar{\boldsymbol{y}}_{j,\rho}^{(s)} - \boldsymbol{m}_{j,\rho}^{(s)})(\bar{\boldsymbol{y}}_{j,\rho}^{(s)} - \boldsymbol{m}_{j,\rho}^{(s)})^T, \tag{31}$$

$$\nu_{j,\rho}^{(s)} = \nu_0 + N_{j,\rho}^{(s)} + 1, \tag{32}$$

and

$$
\begin{aligned}
N_{j,\rho}^{(s)} &= \sum_i \hat{z}_{i,j} N_i^{(s)} \sum_\beta \hat{\nu}^{(s),i,j}(\rho,\beta), \\
\bar{y}_{j,\rho}^{(s)} &= \frac{1}{N_{j,\rho}^{(s)}} \sum_i \hat{z}_{i,j} N_i^{(s)} \sum_\beta \hat{\nu}^{(s),i,j}(\rho,\beta) \boldsymbol{\mu}_{i,\beta}^{(s,b)}, \\
S_{j,\rho}^{(s)} &= \frac{1}{N_{j,\rho}^{(s)}} \sum_i \hat{z}_{i,j} N_i^{(s)} \sum_\beta \hat{\nu}^{(s),i,j}(\rho,\beta) (\boldsymbol{\mu}_{i,\beta}^{(s,b)} - \bar{\boldsymbol{y}}_{j,\rho}^{(s)})(\boldsymbol{\mu}_{i,\beta}^{(s,b)} - \bar{\boldsymbol{y}}_{j,\rho}^{(s)})^T, \\
C_{j,\rho}^{(s)} &= \frac{1}{N_{j,\rho}^{(s)}} \sum_i \hat{z}_{i,j} N_i^{(s)} \sum_\beta \hat{\nu}^{(s),i,j}(\rho,\beta) (\boldsymbol{\Lambda}_{i,\beta}^{(s,b)})^{-1}.
\end{aligned}
$$

Here $\hat{\nu}^{(s),i,j}(\rho,\beta)$ has the same form with that in VHEM algorithm (Coviello et al., 2014), and $N_{j,\rho}^{(s)}$ is the expected number of samples that have been assigned to $\mathcal{R}_j$ with state ρ during the whole time. From (29), as more samples are assigned to $\mathcal{R}_j$ with state ρ (i.e., $N_{j,\rho}^{(s)}$ increases), the $\gamma_{j,\rho}^{(s)}$ will increase and the covariance of posterior of $\boldsymbol{\mu}_{j,\rho}^{(s,r)}$ will decrease; At the same time, the degree of freedom $\nu_{j,\rho}^{(s)}$ will increase, which leads to increasing precision of the posterior of $\boldsymbol{\mu}_{j,\rho}^{(s,r)}$. The update equation of $\boldsymbol{m}_{j,\rho}^{(s)}$ is a mix between the prior and the soft sample mean $\bar{y}_{j,\rho}^{(s)}$. Similarly, the update for $\boldsymbol{W}_{j,\rho}^{(s)}$ is the mix between the prior and the soft sample covariance $S_{j,\rho}^{(s)}$ and mean base covariance $C_{j,\rho}^{(s)}$.

4. $\log q^*(\boldsymbol{\pi}^{(s,r)}, \boldsymbol{A}^{(s,r)})$

$$
\begin{aligned}
\log q^*(\boldsymbol{\pi}^{(s,r)}, \boldsymbol{A}^{(s,r)}) \propto & \sum_s \sum_i \sum_j \hat{z}_{i,j} N_i^{(s)} \sum_{\boldsymbol{\beta}} \pi_{i,\boldsymbol{\beta}}^{(s,b)} \sum_{\boldsymbol{\rho}} \hat{\phi}^{(s),i,j}(\boldsymbol{\rho}|\boldsymbol{\beta}) \Big[\log \pi_{j,\rho_1}^{(s,r)} + \sum_{t=2}^{\tau} \log a_{j,\rho_{t-1},\rho_t}^{(s,r)} \Big] \\
& + \sum_s \sum_j \log p(\boldsymbol{\pi}_j^{(s,r)}, \boldsymbol{A}_j^{(s,r)}) \\
= & \sum_s \sum_j \Big[\log q^*(\boldsymbol{\pi}_j^{(s,r)}) + \log q^*(\boldsymbol{A}_j^{(s,r)}) \Big].
\end{aligned}
$$

For $\log q^*(\boldsymbol{\pi}_j^{(s,r)})$, we have

$$
\begin{aligned}
\log q^*(\boldsymbol{\pi}_j^{(s,r)}) &= \sum_i \hat{z}_{i,j} N_i^{(s)} \sum_{\beta_1} \pi_{i,\beta_1}^{(s,b)} \sum_{\rho_1} \hat{\phi}^{(s),i,j}(\rho_1|\beta_1) \log \pi_{j,\rho_1}^{(s,r)} + \log p(\pi_j^{(s,r)}) \\
&= \sum_i \hat{z}_{i,j} N_i^{(s)} \sum_{\beta_1} \pi_{i,\beta_1}^{(s,b)} \sum_{\rho_1} \hat{\phi}^{(s),i,j}(\rho_1|\beta_1) \log \pi_{j,\rho_1}^{(s,r)} + \sum_{\rho_1} (\eta_0 - 1) \log \pi_{j,\rho_1}^{(s,r)} \\
&= \sum_{\rho_1} (\sum_i \hat{z}_{i,j} N_i^{(s)} \sum_{\beta_1} \pi_{i,\beta_1}^{(s,b)} \hat{\phi}^{(s),i,j}(\rho_1|\beta_1) + \eta_0 - 1) \log \pi_{j,\rho_1}^{(s,r)} \\
&= \sum_{\rho_1} (\eta_{j,\rho_1}^{(s)} - 1) \log \pi_{j,\rho_1}^{(s,r)},
\end{aligned}
$$

where,

$$\eta^{(s)}_{j,\rho_1} = N^{(s)}_{j,\rho_1} + \eta_0, \tag{33}$$

$$N^{(s)}_{j,\rho_1} = \sum_i \hat{z}_{i,j} N^{(s)}_i \hat{\nu}^{(s),i,j}_1(\rho_1). \tag{34}$$

For $\log q^*(\boldsymbol{A}^{(s,r)}_j)$, we have

$$\begin{aligned}
\log q^*(\boldsymbol{A}^{(s,r)}_j) &= \sum_i \hat{z}_{i,j} N^{(s)}_i \sum_{\boldsymbol{\beta}} \pi^{(s,b)}_{i,\boldsymbol{\beta}} \sum_{\boldsymbol{\rho}} \hat{\phi}^{(s),i,j}(\boldsymbol{\rho}|\boldsymbol{\beta}) \Big[\sum_{t=2}^{\tau} \log a^{(s,r)}_{j,\rho_{t-1},\rho_t}\Big] + \log p(\boldsymbol{A}^{(s,r)}_j) \\
&= \sum_\rho \sum_{\rho'} \sum_i \hat{z}_{i,j} N^{(s)}_i \xi^{(s),i,j}(\rho,\rho') \log a^{(s,r)}_{j,\rho,\rho'} + \sum_\rho \sum_{\rho'} (\epsilon_{\rho,0} - 1) \log a^{(s,r)}_{j,\rho,\rho'} \\
&= \sum_\rho \Big[\sum_{\rho'} (\sum_i \hat{z}_{i,j} N^{(s)}_i \xi^{(s),i,j}(\rho,\rho') + \epsilon_{\rho,0} - 1) \log a^{(s,r)}_{j,\rho,\rho'} \Big] \\
&= \sum_\rho \Big[\sum_{\rho'} (\epsilon^{(s)}_{j,\rho,\rho'} - 1) \log a^{(s,r)}_{j,\rho,\rho'} \Big],
\end{aligned}$$

where

$$\epsilon^{(s)}_{j,\rho,\rho'} = N^{(s)}_{j,\rho,\rho'} + \epsilon_{\rho,0}, \tag{35}$$

$$N^{(s)}_{j,\rho,\rho'} = \sum_i \hat{z}_{i,j} N^{(s)}_i \xi^{(s),i,j}(\rho,\rho'). \tag{36}$$

Here $\hat{\nu}^{(s),i,j}_1(\rho_1)$ and $\hat{\xi}^{(s),i,j}(\rho,\rho')$ have the same form as in the VHEM algorithm (Coviello et al., 2014). $N^{(s)}_{j,\rho_1}$ is the number of samples which have been assigned to $\mathcal{R}^{(s)}_j$ and have initial state ρ_1, and $N^{(s)}_{j,\rho,\rho'}$ is the number of samples which have been assigned to $\mathcal{R}^{(s)}_j$ and have transition from state ρ to ρ'.

B.5 Lower Bound

With the optimal variational distribution q^* we now compute the lower bound $\mathcal{L}_{(K^{(r)},S^{(r)})}(q)$, which is used for model selection,

$$\begin{aligned}
\mathcal{L}_{(K^{(r)},S^{(r)})}(q) &= \sum_{\boldsymbol{Z}} \int q(\boldsymbol{Z},\boldsymbol{\Omega},\boldsymbol{\pi},\boldsymbol{A},\boldsymbol{\mu},\boldsymbol{\Lambda}) \log \frac{p(\boldsymbol{Y},\boldsymbol{Z},\boldsymbol{\Omega},\boldsymbol{\pi},\boldsymbol{A},\boldsymbol{\mu},\boldsymbol{\Lambda})}{q(\boldsymbol{Z},\boldsymbol{\Omega},\boldsymbol{\pi},\boldsymbol{\mu},\boldsymbol{\Lambda})} d\boldsymbol{\Omega} d\boldsymbol{\pi} d\boldsymbol{A} d\boldsymbol{\mu} d\boldsymbol{\Lambda} \\
&= \mathbb{E} \log p(\boldsymbol{Y},\boldsymbol{Z},\boldsymbol{\Omega},\boldsymbol{\pi},\boldsymbol{A},\boldsymbol{\mu},\boldsymbol{\Lambda}) - \mathbb{E} \log q(\boldsymbol{Z},\boldsymbol{\Omega},\boldsymbol{\pi},\boldsymbol{A},\boldsymbol{\mu},\boldsymbol{\Lambda}) \\
&= \mathbb{E} \log p(\boldsymbol{Y}|\boldsymbol{Z},\boldsymbol{\pi},\boldsymbol{A},\boldsymbol{\mu},\boldsymbol{\Lambda}) + \mathbb{E} \log p(\boldsymbol{Z}|\boldsymbol{\Omega}) + \mathbb{E} \log p(\boldsymbol{\pi},\boldsymbol{A}) + \mathbb{E} \log p(\boldsymbol{\mu},\boldsymbol{\Lambda}) + \mathbb{E} \log p(\boldsymbol{\Omega}) \\
&\quad - \mathbb{E} \log q(\boldsymbol{Z}) - \mathbb{E} \log q(\boldsymbol{\Omega}) - \mathbb{E} \log q(\boldsymbol{\pi},\boldsymbol{A}) - \mathbb{E} \log q(\boldsymbol{\mu},\boldsymbol{\Lambda}) \qquad (37)
\end{aligned}$$

where, to keep the notation uncluttered, we have omitted the superscript $*$ on the q distributions, superscript (r) on the parameters, along with the subscripts on the expectation operators because each expectation is taken with respect to all of the random variables in its argument. Next, we look at each term,

1. $\mathbb{E}\log p(\boldsymbol{Y}|\boldsymbol{Z},\mathcal{R})$:

$$\mathbb{E}\log p(\boldsymbol{Y}|\boldsymbol{Z},\mathcal{R}) \geq \sum_{s=1}^{S}\sum_{i=1}^{K^{(s,b)}}\sum_{j=1}^{K^{(r)}} \hat{z}_{ij} N_i^{(s)} \tilde{\mathcal{L}}_{HMM}^{(s),i,j}.$$

2. $\mathbb{E}\log p(\boldsymbol{Z}|\boldsymbol{\Omega})$:

$$\mathbb{E}\log p(\boldsymbol{Z}|\boldsymbol{\Omega}) = \sum_{s=1}^{S}\sum_{i=1}^{K^{(s,b)}}\sum_{j=1}^{K^{(r)}} \hat{z}_{ij} N_i^{(s)} \mathbb{E}\log \omega_j = \sum_{j=1}^{K^{(r)}} N_j \log \tilde{\omega}_j.$$

$$\log \tilde{\omega}_j \triangleq \mathbb{E}_{\boldsymbol{\omega}}[\log \omega_j] = \psi(\alpha_j) - \psi(\hat{\alpha})$$

where $\hat{\alpha} = \sum_j \alpha_j$ and $\psi(x)$ is the digamma function.

3. $\mathbb{E}\log p(\boldsymbol{\Omega})$:

$$\mathbb{E}\log p(\boldsymbol{\Omega}) = \log C(\alpha_0) + \sum_j (\alpha_0 - 1)\log \tilde{\omega}_j.$$

4. $\mathbb{E}\log p(\boldsymbol{\pi})$:

$$\begin{aligned}\mathbb{E}\log p(\boldsymbol{\pi}) &= \sum_{s=1}^{S}\sum_{j=1}^{K^{(r)}} \mathbb{E}\log p(\boldsymbol{\pi}_j^{(s)}) \\ &= \sum_{s=1}^{S}\left[K^{(r)}\log C(\eta_0) + \sum_{j=1}^{K^{(r)}}\sum_{\rho}(\eta_0 - 1)\log \tilde{\pi}_{j,\rho}^{(s)}\right].\end{aligned}$$

5. $\mathbb{E}\log p(\boldsymbol{A})$:

$$\begin{aligned}\mathbb{E}\log p(\boldsymbol{A}) &= \sum_{s=1}^{S}\sum_{j=1}^{K^{(r)}}\sum_{\rho} \mathbb{E}\log p(\boldsymbol{a}_{j,\rho}^{(s)}) \\ &= \sum_{s=1}^{S}\left[K^{(r)}\sum_{\rho}\log C(\epsilon_{\rho,0}) + \sum_{j=1}^{K^{(r)}}\sum_{\rho}\sum_{\rho'}(\epsilon_{\rho,0} - 1)\log \tilde{a}_{j,\rho,\rho'}^{(s)}\right].\end{aligned}$$

6. $\mathbb{E}\log p(\boldsymbol{\mu},\boldsymbol{\Lambda})$:

$$\begin{aligned}\mathbb{E}\log p(\boldsymbol{\mu},\boldsymbol{\Lambda}) &= \sum_{j=1}^{K^{(r)}}\sum_{s=1}^{S} \mathbb{E}\log p(\boldsymbol{\mu}_j^{(s)},\boldsymbol{\Lambda}_j^{(s)}) \\ &= \sum_{s=1}^{S}\Bigg\{\frac{1}{2}\sum_{j=1}^{K^{(r)}}\sum_{\rho}\left[d\log\frac{\gamma_0}{2\pi} + \log\tilde{\boldsymbol{\Lambda}}_{j,\rho}^{(s)} - \frac{d\gamma_0}{\gamma_{j,\rho}^{(s)}} - \lambda_0\nu_{j,\rho}^{(s)}(\boldsymbol{m}_{j,\rho}^{(s)} - \boldsymbol{m}_0)^T\boldsymbol{W}_{j,\rho}^{(s)}(\boldsymbol{m}_{j,\rho}^{(s)} - \boldsymbol{m}_0)\right] \\ &\quad + K^{(r)}S^{(r)}\log B(\boldsymbol{W}_0,\nu_0) + \frac{(\nu_0 - d - 1)}{2}\sum_{j=1}^{K^{(r)}}\sum_{\rho}\log\tilde{\boldsymbol{\Lambda}}_{j,\rho}^{(s)} - \frac{1}{2}\sum_{j=1}^{K^{(r)}}\sum_{\rho}\nu_{j,\rho}^{(s)}\,\mathrm{Tr}(\boldsymbol{W}_0^{-1}\boldsymbol{W}_{j,\rho}^{(s)})\Bigg\}.\end{aligned}$$

$$\log \tilde{\mathbf{\Lambda}}_{j,\rho}^{(s)} \triangleq \mathbb{E}_{\mathbf{\Lambda}_{j,\rho}^{(s)}}[\log |\mathbf{\Lambda}_{j,\rho}^{(s)}|]$$
$$= \sum_{i=1}^{D} \psi\Big(\frac{\nu_{j,\rho}^{(s)} + 1 - i}{2}\Big) + D \log 2 + \log |\boldsymbol{W}_{j,\rho}^{(s)}|$$

where D is the dimension of data.

7. $\mathbb{E} \log q(\boldsymbol{Z})$:

$$\mathbb{E} \log q(\boldsymbol{Z}) = \sum_{i=1}^{K^{(b)}} \sum_{j=1}^{K^{(r)}} \hat{z}_{ij} \log \hat{z}_{ij}.$$

8. $\mathbb{E} \log q(\mathbf{\Omega})$:

$$\mathbb{E} \log q(\mathbf{\Omega}) = \log C(\boldsymbol{\alpha}) + \sum_{j} (\alpha_j - 1) \log \tilde{\omega}_j.$$

9. $\mathbb{E} \log q(\boldsymbol{\pi}, \boldsymbol{A})$:

$$\mathbb{E} \log q(\boldsymbol{\pi}, \boldsymbol{A}) = \sum_{j=1}^{K^{(r)}} \sum_{s=1}^{S} \mathbb{E} \log q(\boldsymbol{\pi}_j^{(s)}, \boldsymbol{A}_j^{(s)}) = \sum_{s=1}^{S} \Big\{ \sum_{j=1}^{K^{(r)}} \mathbb{E} \log q(\boldsymbol{\pi}_j^{(s)}) + \sum_{j=1}^{K^{(r)}} \sum_{\rho} \mathbb{E} \log q(\boldsymbol{a}_{j,\rho}^{(s)}) \Big\},$$
$$\mathbb{E} \log q(\boldsymbol{\pi}_j^{(s)}) = \log C(\boldsymbol{\eta}_j^{(s)}) + \sum_{\rho} (\eta_{j,\rho}^{(s)} - 1) \log \tilde{\pi}_{j,\rho}^{(s)},$$
$$\mathbb{E} \log q(\boldsymbol{a}_{j,\rho}^{(s)}) = \log C(\boldsymbol{\epsilon}_{j,\rho}^{(s)}) + \sum_{\rho'} (\epsilon_{j,\rho,\rho'}^{(s)} - 1) \log \tilde{a}_{j,\rho,\rho'}^{(s)}.$$

10. $\mathbb{E} \log q(\boldsymbol{\mu}, \mathbf{\Lambda})$:

$$\mathbb{E} \log q(\boldsymbol{\mu}, \mathbf{\Lambda}) = \sum_{j=1}^{K^{(r)}} \sum_{s=1}^{S} \mathbb{E} \log q(\boldsymbol{\mu}_{j,\rho}^{(s)}, \mathbf{\Lambda}_{j,\rho}^{(s)})$$
$$= \sum_{s=1}^{S} \sum_{j=1}^{K^{(r)}} \sum_{\rho} \Big[\frac{1}{2} \log \tilde{\mathbf{\Lambda}}_{j,\rho}^{(s)} + \frac{d}{2} \log \frac{\gamma_{j,\rho}^{(s)}}{2\pi} - \frac{d}{2} - H[q(\mathbf{\Lambda}_{j,\rho}^{(s)})] \Big]$$

where $H[q(\mathbf{\Lambda}_{j,\rho}^{(s)})]$ is the entropy of the Wishart distribution.

B.6 Lower bound derivatives

To obtain the hyperparameters of the prior distributions, we maximize the lower bound w.r.t. the hyperparameters. This is performed using gradient ascent, which requires derivatives of $\mathcal{L}$ w.r.t. each hyperparameter.

1. Derivatives w.r.t. α_0

$$\begin{aligned}
\frac{\partial \mathcal{L}}{\partial \alpha_0} &= \frac{\partial}{\partial \alpha_0}\mathbb{E}[\log p(\boldsymbol{\Omega}^{(r)})] \\
&= \frac{\partial}{\partial \alpha_0}\Big[\log C(\alpha_0) + \sum_j (\alpha_0 - 1)\log \tilde{\omega}_j^{(r)}\Big] \\
&= \frac{\partial}{\partial \alpha_0}\Big[\log C(\alpha_0)\Big] + \sum_j \log \tilde{\omega}_j^{(r)}, \\
\frac{\partial}{\partial \alpha_0}\Big[\log C(\alpha_0)\Big] &= K^{(r)}\psi(K^{(r)}\alpha_0) - K^{(r)}\psi(\alpha_0).
\end{aligned}$$

2. Derivatives w.r.t. η_0

$$\begin{aligned}
\frac{\partial \mathcal{L}}{\partial \eta_0} &= \frac{\partial}{\partial \eta_0}\mathbb{E}[\log p(\boldsymbol{\pi})] \\
&= \frac{\partial}{\partial \eta_0}\sum_{j=1}^{K^{(r)}}\sum_{s=1}^{S}\Big[\log C(\eta_0) + \sum_\rho (\eta_0 - 1)\log \tilde{\pi}_{j,\rho}^{(s,r)}\Big] \\
&= K^{(r)}S\frac{\partial}{\partial \eta_0}\big[\log C(\eta_0)\big] + \sum_s\sum_j\sum_\rho \log \tilde{\pi}_{j,\rho}^{(s,r)}, \\
\frac{\partial}{\partial \eta_0}\Big[\log C(\eta_0)\Big] &= S^{(r)}\psi(S^{(r)}\eta_0) - S^{(r)}\psi(\eta_0).
\end{aligned}$$

3. Derivatives w.r.t. ϵ_0 Here we assume that $\epsilon_{\rho,0} = \epsilon_0, \forall \rho$,

$$\begin{aligned}
\frac{\partial \mathcal{L}}{\partial \epsilon_0} &= \sum_{j=1}^{K^{(r)}}\sum_{s=1}^{S}\sum_\rho \frac{\partial \mathcal{L}}{\partial \epsilon_0}\Big[\mathbb{E}\log p(\boldsymbol{a}_{j,\rho}^{(s,r)})\Big] \\
&= \sum_{j=1}^{K^{(r)}}\sum_{s=1}^{S}\sum_\rho \frac{\partial \mathcal{L}}{\partial \epsilon_0}\Big[\log C(\epsilon_0) + \sum_{\rho'} (\epsilon_0 - 1)\log \tilde{a}_{j,\rho,\rho'}^{(s,r)}\Big] \\
&= K^{(r)}SS^{(r)}\frac{\partial}{\partial \epsilon_0}\big[\log C(\epsilon_0)\big] + \sum_j^{K^{(r)}}\sum_{s=1}^{S}\sum_{\rho,\rho'} \log \tilde{a}_{j,\rho,\rho'}^{(s,r)}, \\
\frac{\partial}{\partial \epsilon_0}\Big[\log C(\epsilon_0)\Big] &= S^{(r)}\psi(S^{(r)}\epsilon_0) - S^{(r)}\psi(\epsilon_0).
\end{aligned}$$

4. Derivatives w.r.t. ν_0

$$\frac{\partial \mathcal{L}}{\partial \nu_0} = \frac{\partial}{\partial \nu_0}\Big[\mathbb{E}\log p(\boldsymbol{\mu}, \boldsymbol{\Lambda})\Big]$$

$$= \frac{\partial}{\partial \nu_0}\Bigg\{\frac{1}{2}\sum_j\sum_s\sum_\rho \Big[d\log\frac{\lambda_0}{2\pi} + \log\tilde{\Lambda}^{(s)}_{j,\rho} - \frac{d\lambda_0}{\lambda^{(s)}_{j,\rho}} - \lambda_0\nu^{(s)}_{j,\rho}(\boldsymbol{m}^{(s)}_{j,\rho} - \boldsymbol{m}_0)^T\boldsymbol{W}^{(s)}_{j,\rho}(\boldsymbol{m}^{(s)}_{j,\rho} - \boldsymbol{m}_0)\Big]$$

$$+ K^{(r)}SS^{(r)}\log B(\boldsymbol{W}_0, \nu_0) + \frac{(\nu_0 - d - 1)}{2}\sum_{j=1}^{K^{(r)}}\sum_s\sum_\rho \log\tilde{\Lambda}^{(s)}_{j,\rho} - \frac{1}{2}\sum_{j=1}^{K^{(r)}}\sum_s\sum_\rho \nu^{(s)}_{j,\rho}\operatorname{Tr}(\boldsymbol{W}_0^{-1}\boldsymbol{W}^{(s)}_{j,\rho})\Bigg\}$$

$$= K^{(r)}SS^{(r)}\frac{\partial}{\partial \nu_0}\log B(\boldsymbol{W}_0, \nu_0) + \frac{1}{2}\sum_{j=1}^{K^{(r)}}\sum_s\sum_\rho \log\tilde{\Lambda}^{(s)}_{j,\rho},$$

$$\frac{\partial}{\partial \nu_0}\log B(\boldsymbol{W}_0, \nu_0) = -\frac{1}{2}\log|\boldsymbol{W}_0| - \frac{d}{2}\log 2 - \frac{1}{2}\sum_{k=1}^{d}\psi(\frac{\nu_0 + 1 - k}{2}).$$

5. Derivatives w.r.t. λ_0

$$\frac{\partial \mathcal{L}}{\partial \lambda_0} = \frac{\partial}{\partial \lambda_0}\Big[\mathbb{E}\log p(\boldsymbol{\mu}, \boldsymbol{\Lambda})\Big]$$

$$= \frac{\partial}{\partial \lambda_0}\Bigg\{\frac{1}{2}\sum_j\sum_s\sum_\rho \Big[d\log\frac{\lambda_0}{2\pi} + \log\tilde{\boldsymbol{\Lambda}}^{(s)}_{j,\rho} - \frac{d\lambda_0}{\lambda^{(s)}_{j,\rho}} - \lambda_0\nu^{(s)}_{j,\rho}(\boldsymbol{m}^{(s)}_{j,\rho} - \boldsymbol{m}_0)^T\boldsymbol{W}^{(s)}_{j,\rho}(\boldsymbol{m}^{(s)}_{j,\rho} - \boldsymbol{m}_0)\Big]$$

$$+ K^{(r)}SS^{(r)}\log B(\boldsymbol{W}_0, \nu_0) + \frac{(\nu_0 - d - 1)}{2}\sum_{j=1}^{K^{(r)}}\sum_s\sum_\rho \log\tilde{\boldsymbol{\Lambda}}^{(s)}_{j,\rho} - \frac{1}{2}\sum_{j=1}^{K^{(r)}}\sum_s\sum_\rho \nu^{(s)}_{j,\rho}\operatorname{Tr}(\boldsymbol{W}_0^{-1}\boldsymbol{W}^{(s)}_{j,\rho})\Bigg\}$$

$$= \frac{1}{2}\sum_{j=1}^{K^{(r)}}\sum_s\sum_\rho \frac{\partial}{\partial \lambda_0}\Big[d\log\frac{\lambda_0}{2\pi} - \frac{d\lambda_0}{\lambda^{(s)}_{j,\rho}} - \lambda_0\nu^{s}_{j,\rho}(\boldsymbol{m}^{(s)}_{j,\rho} - \boldsymbol{m}_0)^T\boldsymbol{W}^{(s)}_{j,\rho}(\boldsymbol{m}^{(s)}_{j,\rho} - \boldsymbol{m}_0)\Big]$$

$$= \sum_s\frac{1}{2}\sum_{j=1}^{K^{(r)}}\sum_\rho \Big[\frac{d}{\lambda_0} - \frac{d}{\lambda^{(s)}_{j,\rho}} - \nu^{(s)}_{j,\rho}(\boldsymbol{m}^{(s)}_{j,\rho} - \boldsymbol{m}_0)^T\boldsymbol{W}^{(s)}_{j,\rho}(\boldsymbol{m}^{(s)}_{j,\rho} - \boldsymbol{m}_0)\Big].$$

6. Derivatives w.r.t. $\boldsymbol{W}_0$

Similar to VBHMM, we assume $\boldsymbol{W}_0$ is a full positive-definite matrix.

$$\frac{\partial \mathcal{L}}{\partial \boldsymbol{W}_0} = \frac{\partial}{\partial \boldsymbol{W}_0}\Bigg\{\frac{1}{2}\sum_j\sum_s\sum_\rho \Big[d\log\frac{\lambda_0}{2\pi} + \log\tilde{\boldsymbol{\Lambda}}^{(s)}_{j,\rho} - \frac{d\lambda_0}{\lambda^{(s)}_{j,\rho}} - \lambda_0\nu^{(s)}_{j,\rho}(\boldsymbol{m}^{(s)}_{j,\rho} - \boldsymbol{m}_0)^T\boldsymbol{W}^{(s)}_{j,\rho}(\boldsymbol{m}^{(s)}_{j,\rho} - \boldsymbol{m}_0)\Big]$$

$$+ K^{(r)}SS^{(r)}\log B(\boldsymbol{W}_0, \nu_0) + \frac{(\nu_0 - d - 1)}{2}\sum_{j=1}^{K^{(r)}}\sum_s\sum_\rho \log\tilde{\boldsymbol{\Lambda}}^{(s)}_{j,\rho} - \frac{1}{2}\sum_{j=1}^{K^{(r)}}\sum_s\sum_\rho \nu^{(s)}_{j,\rho}\operatorname{Tr}(\boldsymbol{W}_0^{-1}\boldsymbol{W}^{(s)}_{j,\rho})\Bigg\}$$

$$= \sum_s\Big[K^{(r)}S^{(r)}\frac{\partial}{\partial \boldsymbol{W}_0}\log B(\boldsymbol{W}_0, \nu_0) - \frac{1}{2}\sum_{j=1}^{K^{(r)}}\sum_\rho \nu^{(s)}_{j,\rho}\frac{\partial}{\partial \boldsymbol{W}_0}\operatorname{Tr}(\boldsymbol{W}_0^{-1}\boldsymbol{W}^{(s)}_{j,\rho})\Big],$$

where

$$\frac{\partial}{\partial \boldsymbol{W}_0} \log B(\boldsymbol{W}_0, \nu_0) = \frac{\partial}{\partial \boldsymbol{W}_0} \log B(\boldsymbol{W}_0, \nu_0) \Big[\frac{\nu_0}{2} \log |\boldsymbol{W}_0| \Big] = -\frac{\nu_0}{2} \boldsymbol{W}_0^{-1},$$

$$\frac{\partial}{\partial \boldsymbol{W}_0} \operatorname{Tr}(\boldsymbol{W}_0^{-1} \boldsymbol{W}_{j,\rho}^{(s)}) = -\boldsymbol{W}_0^{-1} \boldsymbol{W}_{j,\rho}^{(s)} \boldsymbol{W}_0^{-1}.$$

Next, we assume simpler forms of $\boldsymbol{W}_0$

- *Isotropic*: We assume $\boldsymbol{W}_0 = w_0 I$, where $w_0 > 0$ is the parameter. Then,

$$\frac{\partial}{\partial w_0} \log B(\boldsymbol{W}_0, \nu_0) = -\frac{\nu_0 d}{2 w_0},$$

$$\frac{\partial}{\partial w_0} \operatorname{Tr}(\boldsymbol{W}_0^{-1} \boldsymbol{W}_{j,\rho}^{(s)}) = -\frac{1}{w_0^2} \operatorname{Tr}(\boldsymbol{W}_{j,\rho}^{(s)}).$$

- *Diagonal*: We assume $\boldsymbol{W}_0 = \operatorname{diag}(w_0)$, where $w_0 > 0$ is a parameter vector. Then,

$$\frac{\partial}{\partial \boldsymbol{w}_0} \log B(\boldsymbol{W}_0, \nu_0) = -\frac{\nu_0}{2} \frac{1}{w_0},$$

$$\frac{\partial}{\partial \boldsymbol{w}_0} \operatorname{Tr}(\boldsymbol{W}_0^{-1} \boldsymbol{W}_{\rho}^{j}) = -\frac{1}{w_0^2} \operatorname{diag}(\boldsymbol{W}_{j,\rho}^{(s)}).$$

  where operations here are using element-wise product and division.

7. Derivatives w.r.t. $\boldsymbol{m}_0$

$$\frac{\partial \mathcal{L}}{\partial \boldsymbol{m}_0} = \frac{\partial}{\partial \boldsymbol{m}_0} \Bigg\{ \frac{1}{2} \sum_j \sum_s \sum_\rho \Big[d \log \frac{\lambda_0}{2\pi} + \log \tilde{\boldsymbol{\Lambda}}_{j,\rho}^{(s)} - \frac{d\lambda_0}{\lambda_{j,\rho}^{(s)}} - \lambda_0 \nu_{j,\rho}^{(s)} (\boldsymbol{m}_{j,\rho}^{(s)} - \boldsymbol{m}_0)^T \boldsymbol{W}_{j,\rho}^{(s)} (\boldsymbol{m}_{j,\rho}^{(s)} - \boldsymbol{m}_0) \Big]$$

$$+ K^{(r)} S S^{(r)} \log B(\boldsymbol{W}_0, \nu_0) + \frac{(\nu_0 - d - 1)}{2} \sum_{j=1}^{K^{(r)}} \sum_s \sum_\rho \log \tilde{\boldsymbol{\Lambda}}_{j,\rho}^{(s)} - \frac{1}{2} \sum_{j=1}^{K^{(r)}} \sum_s \sum_\rho \nu_{j,\rho}^{(s)} \operatorname{Tr}(\boldsymbol{W}_0^{-1} \boldsymbol{W}_{j,\rho}^{(s)}) \Bigg\}$$

$$= \frac{1}{2} \sum_j \sum_s \sum_\rho \frac{\partial}{\partial \boldsymbol{m}_0} \Big[-\lambda_0 \nu_{j,\rho}^{(s)} (\boldsymbol{m}_{j,\rho}^{(s)} - \boldsymbol{m}_0)^T \boldsymbol{W}_{j,\rho}^{(s)} (\boldsymbol{m}_{j,\rho}^{(s)} - \boldsymbol{m}_0) \Big]$$

$$= \sum_s \sum_j \sum_\rho \lambda_0 \nu_{j,\rho}^{(s)} \boldsymbol{W}_{j,\rho}^{(s)} (\boldsymbol{m}_{j,\rho}^{(s)} - \boldsymbol{m}_0).$$

References

Christopher M Bishop. *Pattern recognition and machine learning.* springer, 2006.

Emanuele Coviello, Antoni B. Chan, and Gert R. G. Lanckriet. Clustering hidden Markov models with variational HEM. *J. Mach. Learn. Res.*, 15(1):697–747, January 2014.
